# Restrictive Fluid Administration vs. Standard of Care in Emergency Department Sepsis Patients (REFACED Sepsis)—protocol for a multicenter, randomized, clinical, proof-of-concept trial

**DOI:** 10.1186/s40814-022-01034-y

**Published:** 2022-03-29

**Authors:** Marie Kristine Jessen, Lars Wiuff Andersen, Marie-Louise Holm Thomsen, Peter Kristensen, Wazhma Hayeri, Ranva Espegård Hassel, Anders Perner, Jens Aage Kølsen Petersen, Hans Kirkegaard

**Affiliations:** 1 Research Center for Emergency Medicine, Department of Clinical Medicine, Aarhus University and Aarhus University Hospital, Palle Juul-Jensens Boulevard 99, J103, DK-8200 Aarhus N, Denmark; 2 Department of Emergency Medicine, Aarhus University Hospital, Aarhus N, Denmark; 3 Department of Anesthesiology and Intensive Care, Aarhus University Hospital, Aarhus N, Denmark; 4 Prehospital Emergency Medical Services, Central Denmark Region, Aarhus N, Denmark; 5 Department of Emergency Medicine, Regional Hospital Viborg, Viborg, Denmark; 6 Department of Emergency Medicine, Regional Hospital Randers, Randers, Denmark; 7 Department of Intensive Care, Copenhagen University Hospital, Rigshospitalet, Copenhagen, Denmark

**Keywords:** Emergency department, Fluid therapy, Sepsis, Infection

## Abstract

**Background:**

Intravenous fluids are often used in the treatment of sepsis. The better strategy regarding fluid volume is debated, but preliminary data in patients with septic shock or sepsis-related hypotension favor restrictive fluid administration. We describe the protocol and statistical analysis plan for the Restrictive Fluid Administration vs. Standard of Care in Emergency Department Sepsis Patients (REFACED Sepsis)—a multicenter, randomized clinical proof-of-concept trial. The aim of the REFACED Sepsis trial is to test if a restrictive intravenous fluid protocol in emergency department patients with sepsis without shock is feasible and decreases the intravenous fluid volume administered in comparison to standard care.

**Methods:**

This is an investigator-initiated, multicenter, randomized, parallel-group, open-labeled, feasibility trial investigating volumes of crystalloid fluid within 24 h in 124 patients with sepsis without shock enrolled at three emergency departments in the Central Denmark Region. Patients are allocated to two different intravenous fluid regimens: a restrictive approach using four trigger criteria for fluid administration vs. standard care.

The primary, feasibility outcome is total intravenous, crystalloid fluid volume within 24 h, and key secondary outcomes include protocol violations, total fluids (intravenous and oral) within 24 h, and serious adverse reactions and suspected unexpected serious adverse reactions.

Status: The trial started in November 2021, and the last patient is anticipated to be included in January 2022.

**Discussion:**

Sepsis is very common in emergency department patients and fluid administration is very frequently administered in these patients. However, the evidence to guide fluid administration is very sparse. This feasibility trial will be the foundation for a potential future large-scale trial investigating restrictive vs. standard fluid administration in patients with sepsis.

**Trial registration:**

EudraCT number: 2021-000224-35 (date: 2021 May 03), ClinicalTrials.gov number: NCT05076435 (date: 2021 October 13), Committee on Health Research Ethics – Central Denmark Region: 1-10-72-163-21 (date: 2021 June 28).

**Supplementary Information:**

The online version contains supplementary material available at 10.1186/s40814-022-01034-y.

## Introduction

Sepsis is common and accounts for approximately a quarter of all admissions from Danish medical emergency departments (ED) [1] and for more than 500,000 yearly patient visits in the USA [2]. Sepsis patients are at risk of disease progression to septic shock (incidences from 7 to 26% [3–6]) with a high mortality [7–9]. Also, the morbidity and effect on health-related quality of life after sepsis is significant [10–12].

The mainstay of sepsis treatment in the initial phase includes intravenous antibiotics and fluids, source control, and supportive care, if necessary [13]. The effects of fluid resuscitation in sepsis are uncertain, and liberal administration may lead to overhydration and edema [9, 14–24]. The choices around fluid volumes are supported by low quality of evidence, which may contribute to marked practice variation [9, 25–29]. The international Surviving Sepsis Campaign (SSC) guides the treatment of sepsis associated hypotension [30], but does not give any recommendations for fluids in sepsis patients not in shock, despite the fact that sepsis is almost 60 times more frequent then septic shock [1]. Evidence within the field has been requested by experts [31, 32].

Recently, there has been a number of observational studies and interventional trials on fluid volumes in septic shock and several randomized trials are ongoing [15, 26, 33, 34]. In a randomized pilot trial, it was possible to reduce volumes of resuscitation fluids with a restrictive fluid protocol in septic shock patients in the intensive care unit (ICU) [33]. In another pilot trial in the ED by Macdonald et al., it was possible to reduce fluids with 30% with a fluid restrictive and early vasopressor approach in patients with sepsis associated hypotension [34]. A systematic review with meta-analysis of all randomized trials found no statistically significant difference between lower vs. higher fluid volumes in all-cause mortality in patients with sepsis, but the point estimates did favor fluid restriction for all outcomes. Six of seven trials in the review were done in the ICU setting [35].

The aim of the Restrictive Fluid Administration vs. Standard of Care in Emergency Department Sepsis Patients (REFACED Sepsis) trial is to test if a restrictive intravenous fluid protocol in ED patients with sepsis without shock is feasible and if the treatment protocol decreases the volumes of intravenous fluid administered in comparison to standard care. Should the trial prove feasible with separation between the two interventions, a large-scale trial assessing patient important outcomes is warranted.

## Methods

### Protocol

The full protocol is provided in the Supplemental material and all previous versions are available on the trial website [36]. The protocol was developed in accordance with the International Conference on Harmonization (ICH) guidelines [37–39] and the Standard Protocol Items: Recommendations for Interventional Trials (SPIRIT) statement [40, 41]. The trial was registered at the EU Clinical Trials Register (EudraCT number: 2021-000224-35 (date: 2021 May 03)) and at ClinicalTrials.gov (Identifier: NCT05076435 (date: 2021 October 13)). The trial protocol was approved by the Committee on Health Research Ethics—Central Denmark Region (Identifier: 1-10-72-163-21 (date: 2021 June 28)).

### Trial design

The REFACED Sepsis trial is an investigator-initiated, multicenter, randomized, parallel-group, open-labeled, feasibility trial, randomizing 124 sepsis patients without shock to a restrictive approach for fluid administration within the first 24 h or standard care.

### Setting

The trial will be conducted at the EDs at Aarhus University Hospital and the Regional Hospitals in Randers and Viborg, Denmark.

### Eligibility

We will include patients fulfilling all of the following inclusion criteria: (1) unplanned emergency department admission, (2) age ≥ 18 years, (3) sepsis defined as (a) suspected infection by the treating clinician AND (b) blood cultures drawn AND (c) intravenous antibiotics administered or planned AND (d) an infection-related increase of SOFA-score ≥ 2 [42], and (4) expected hospital stay > 24 h as deemed by the treating clinician. We will exclude all patients fulfilling any of the following exclusion criteria: (1) ≥ 500 ml of intravenous fluids given prior to randomization, (2) use of invasive ventilation or vasopressor at the time of screening, (3) known or suspected severe bleeding as per the treating clinician, (4) known or suspected pregnancy (women aged < 45 years will have a pregnancy test performed before enrollment), (5) prior enrollment in the trial, and (6) patients, who the clinician expect not to survive the next 24 h. Patients will be included irrespective of COVID-19 status, since inclusion and randomization often will occur prior to test results. According to Danish law, it is only allowed to include patients in a study who can either all provide written, informed consent OR patients who are *not* able to provide written consent after being given oral and written trial information before randomization. Since most sepsis patients are not able to provide informed consent, we decided to only include patients who cannot provide written, informed consent at inclusion. This means that all included patients will be either severely in pain, distress, confused, delirious, respiratory, or circulatory acutely affected and/or ill. The inclusion will be approved through legal guardian informed consent from an independent physician. For further details on ethics and consent, see the “Ethical considerations and consent” section.

### Intervention

**Fig. 1:**
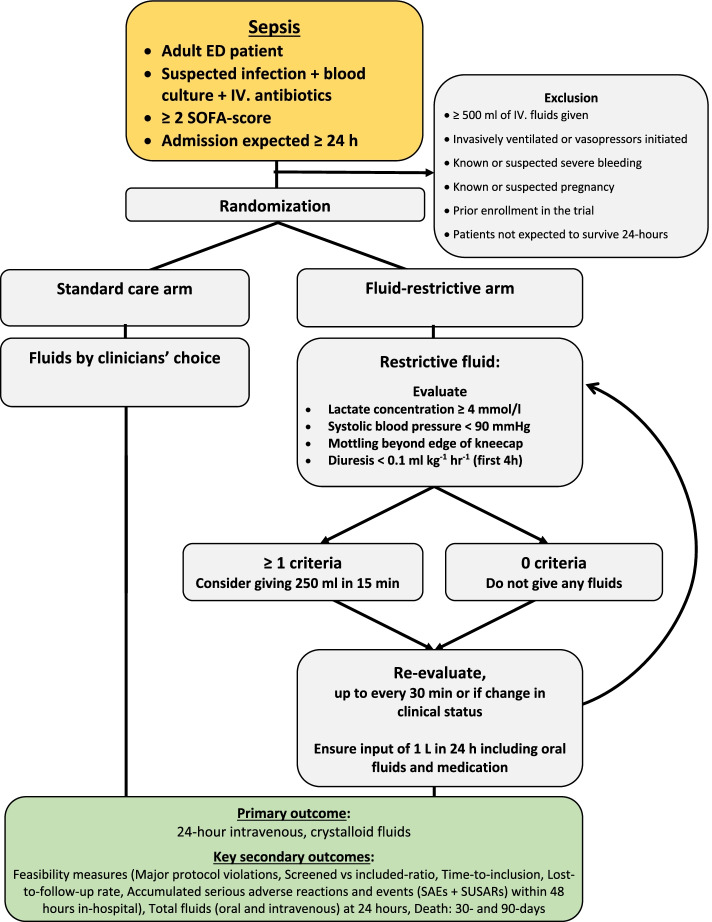
Inclusion criteria, exclusion criteria and treatment algorithms

Patients are randomized to either standard care or restrictive fluid administration for 24 h by the treating physician or the treating physician in collaboration with research assistants. The intervention protocols in the REFACED Sepsis trial are targeted intravenous crystalloid fluid. Fluid restriction vs. standard care fluid therapy cannot be blinded for investigators, clinical staff, or participants. The two treatment algorithms are shown in Fig. 1 and described further in the following.

#### Standard care

Intravenous fluid will be given as per choice of the treating team. There will be no lower or upper limit for the use of either intravenous or oral/enteral fluids.

#### Restrictive fluid administration

No intravenous crystalloid fluids should be given unless one of the below mentioned situations occur:

A fluid bolus of 250 ml isotonic crystalloid (normal saline or Ringer’s acetate/lactate) may be given within 15 min if one or more of the following occurs (hypoperfusion criteria):

- Lactate concentration ≥ 4 mmol/l (arterial or venous)
- Hypotension (systolic blood pressure < 90 mmHg)
- Mottling beyond edge of kneecap (i.e., Mottling score > 2) [43]
- Severe oliguria, i.e., diuresis < 0.1 ml/kg/h, during the first 4 h of admission

After 30 min, the effect of a fluid bolus may be assessed by re-evaluation of the four hypoperfusion criteria mentioned above by the treating clinician. If one or more of the criteria are still fulfilled, a fluid bolus as defined above may be repeated. This circle of a fluid bolus, 30 min and re-evaluation may be repeated as long as clinically indicated. At any time, the clinician can start vasopressors if deemed necessary.

The treating physician may at any time violate the protocol by giving fluid although none of the above-mentioned criteria are fulfilled if found to be in the interest of the patient. The physician must state the reason for violating the protocol.

Intravenous fluids may be given as carrier for medications, but the volume should be reduced to the lowest possible volume for the given medication. In case of documented overt fluid losses (e.g., vomiting, large aspirates, diarrhea, drain losses, or ascites drainage), intravenous fluid may be given to correct for the loss. In case the oral/enteral route for water or electrolyte solutions is contraindicated or has failed as judged by the clinical team, intravenous fluids may be given to:

- Correct significant electrolyte deficiencies
- Ensure a total fluid input of 1 L per 24 h (fluids with medications and nutrition count as input)

If a patient undergoes surgery during the 24-h inclusion period, they temporarily pause the protocol, but clinicians will be encouraged to continue restrictive fluid therapy.

Research assistants (medical students) will be present at all three sites from 14.00-23.30 to ensure enrollment of the patients, information to the involved clinical teams and encourage to complete documentation of all fluids.

### Randomization

Eligible patients fulfilling all inclusion criteria and no exclusion criteria will be randomized 1:1 using a centralized web-based system according to a computer-generated allocation sequence list, with varying block sizes, stratified for site. The allocation sequence list and block sizes are only known by the data manager at Trial Partner® at Aarhus University and remains concealed from the investigators until the last patient has completed the study.

### Outcomes

The primary outcome will be the total volume of all administered intravenous, crystalloid fluids within 24 h of randomization.

The secondary outcomes will be as follows: feasibility measures (number of patients with major protocol violations, number of patients screened positive (i.e., with all inclusion criteria fulfilled and no exclusion criteria fulfilled) vs randomized, time from admission to inclusion, number of patients with incomplete data on the primary outcome 24-h fluids (e.g. due to discharge within 24 h or in hospital death)), accumulated serious adverse reactions and events within 7 days in-hospital, and total fluids (oral, total intravenous, and total oral and intravenous) at 24 h. We will also report in-hospital mortality and 30- and 90-day mortality, in-hospital length of stay (LOS), mechanical ventilation within 7 days of admission (yes/no), vasopressor use within 7 days of admission (yes/no), and development or worsening of acute kidney failure according to the KDIGO3-criteria within 7 days of admission [44].

### Sample size

The trial was powered to the primary outcome of 24-h total intravenous fluids. The sample size calculation is based on data from an observational study conducted in the Central Denmark Region in which sepsis patients meeting inclusion criteria for the current trial received 2670 ml intravenous fluids (SD 1695) [29]. We therefore estimated that the total amount of IV fluid in the control group will be 2650 ml (standard deviation 1700 ml). We consider a mean difference of 1 L to be clinical meaningful and therefore estimate 1650 ml (standard deviation 1.7 L) in the intervention group. Based on these estimates, an alpha of 5%, a power of 90%, and a two-sample *t*-test, a sample size of 124 patients is required; 62 in each treatment arm.

### Statistical analysis plan

The statistical reporting will adhere to the CONSORT guidelines [45, 46]. All tests will be two-sided, a *p*-value < 0.05 will be considered statistically significant, and 95% confidence intervals will be presented.

All analyses will be conducted in a modified intention-to-treat (ITT) population defined as all randomized participants for whom consent to use data was obtained. We will perform the primary analysis adjusted for the stratification variable, trial site. The two groups will be compared regarding baseline characteristics using descriptive statistics.

To estimate the mean difference in fluid volume between groups, we will use linear regression with adjustment for the stratification variable to see if it is feasible to reach 1000 ml difference. If the data is severely non-normally distributed, we will consider other appropriate options (e.g., “robust standard errors” or transformation). Other continuous variables will be analyzed similarly. For binary outcomes, we will use logistic regression adjusted for site and results will be presented as odds ratios.

Missing data will be reported. We do not expect any missing data for the primary outcome (except for those discharged or dying within the 24 h) or the key secondary outcomes. Patients discharged within 24 h or who died within 24 h, will be included in the analysis with the volume of fluid they received until discharge/death. We do not expect missing data on mortality or adverse events. Multiple imputation using known risk factors for outcomes in sepsis will be used to impute values for patients with missing data if missing data is substantial (> 10%). There will be no predefined stopping criteria for this feasibility trial. All analyses will be performed using Stata version 17 (StataCorp LP, College Station, TX, USA).

### Data collection and follow up

The treating team will register limited data during the randomization process, i.e., patient identifier (i.e., Danish Central Personal Register number), site, and inclusion/exclusion criteria. A paper case report form (CRF) (a bedside REFACED Sepsis resuscitation chart) for collecting data on fluid management will be placed at the patient’s bedside. The paper CRF will be filled out by the treating team and/or research assistants during the 24 h; timing of fluids administered, indication for fluid bolus, fluid type and volume and time to re-evaluation and protocol violations and reasons for these. Oral and other intravenous fluid administrations will be noted on the CRF. The research assistants are not allowed to administer/prescribe any fluids. Further data will be obtained from the electronic medical record by the research team including vital signs, blood tests, and further clinical data; all data will be based on measurements and assessments made by the clinical team. A trained member of the research team will be responsible for data collection and entry into the eCRF from the electronic medical journal and from the paper-CRF. Data will be entered directly into the REDCap database from the electronic medical journal. Details of the included variables are provided in the full protocol.

### Clinical treatment

The clinical management of included patients, other than fluid strategy according to randomization, will be at the complete discretion of the treating clinical team in order to test the interventions in a real-life clinical scenario.

### Ethical considerations and consent

A detailed description of the ethical considerations is provided in the protocol in the supplemental material. The trial was approved by the regional ethics committee (case number: 1-10-72-163-21 on June 28, 2021, and Danish Medicine Agency (EudraCT number: 2021-000224-35) on March 05, 2021. Consent is obtained according to Danish law using a two-to-three step approach. Before randomization, verbal, and subsequent written, consent for enrollment is obtained by research staff from an independent physician (first trial guardian). Second, after randomization, consent is obtained from the patient if the patient has regained ability to fully understand the trial circumstances and give written consent. If the patient is still not able to give written consent, informed consent is obtained from either a surrogate/next of kin or the treating physician (second trial guardian). If so, consent is later obtained from the patient as soon as feasible if full ability to provide written consent is regained.

The trial is monitored according to the standards of Good Clinical Practice [47]. The study will be conducted independent of the financial sponsors and the financial sponsors have no role in design, conduct, or reporting the findings of the study.

### Data sharing

Six months after the publication of the last results, all de-identified individual patient data will be made available for data sharing [48]. Procedures, including re-coding of key variables, will be put in place to allow for complete de-identification of the data.

## Discussion

The current article describes the design of the REFACED Sepsis trial, a feasibility trial investigating if a fluid restrictive protocol reduces 24-h crystalloid fluids in sepsis patients without shock admitted to the emergency department in comparison to standard care.

The optimal intravenous fluid strategy in adults with sepsis without shock is unknown. In septic shock, trials on restrictive vs. liberal fluid administration have pointed towards benefit with fluid restriction, although the evidence is still uncertain. Three large-scale studies are currently investigating fluids in septic shock and sepsis-associated hypotension. The CLASSIC-trial (ClinicalTrials.gov Identifier: NCT03668236, just finished enrollment) enrolling septic shock patients in the ICU in primarily Europe [49]. The ARISE-FLUIDS (ClinicalTrials.gov Identifier: NCT04569942) enrolling ED patients with sepsis-associated hypotension in Australia and New Zealand [34]. And lastly, the CLOVERS trial (ClinicalTrials.gov Identifier: NCT03434028) is currently randomizing septic shock patients in the ED to either 24 h liberal fluids or restrictive fluids in the USA [50]. With existing data and those from the ongoing randomized trials primarily in septic shock/sepsis-associated hypotension, the REFACED Sepsis trial will provide important knowledge on the use of intravenous fluid volumes in adult ED patients with sepsis without shock at admission with a possibility of improving care to a large proportion of ED patients.

The primary outcome of the current feasibility trial is 24-h intravenous crystalloid fluid administration. This was chosen to determine if it is feasible to reduce fluid volumes using a restrictive fluid administration protocol. 24 h-fluids were chosen since it represents the most critical part of the resuscitative phase of the sepsis incident. Twenty-four hours of intervention is also used in the CLOVERS trial [50] and up to 24 h in the ARISE FLUIDS study [34]. Secondary outcomes and feasibility measures have been included to be able to conduct a power-calculation for a future large-scale trial. The study is not powered to investigate differences in outcomes other than 24-h fluid administration.

The four hypoperfusion criteria for administration of crystalloid fluids in the restrictive arm were chosen to represent central (systolic blood pressure), general (lactate), peripheral (mottling), and renal (oliguria) circulation and perfusion with inspiration from the CLASSIC trial [33, 49].

The trial design is pragmatic regarding all other aspects of treatments, including fluid type, encouraging the clinical team to follow routine practice to be able to increase external validity as much as possible. The REFACED Sepsis trial intervention cannot be masked for investigators, clinicians, research assistants nor patients, as blinding of the two fluid strategies is not feasible in clinical practice. The non-blinded design increases the risk of bias [51]. We expect that clinicians will violate the protocol, probably most often in patients in the restrictive study arm. Despite protocol violations, a difference in resuscitation fluid volumes in the CLASSIC pilot trial was observed without any safety concerns [33].

### Status

The trial was initiated on November 3, 2021, and is expected to be completed within 2–3 months.

## Supplementary Information

**Additional file 1.**

## Data Availability

Six months after the publication of the last results, all de-identified individual patient data will be made available for data sharing [48]. Procedures, including re-coding of key variables, will be put in place to allow for complete de-identification of the data.

## Abbreviations

CONSORT: Consolidated Standards of Reporting Trials
CRF: Case report form
ED: Emergency department
ICU: Intensive care unit
ICH: International Conference on Harmonization
ITT: Intention to treat
KDIGO3: Kidney Disease: Improving Global Outcomes 3
SOFA-score: Sequential Organ Failure Assessment (SOFA) Score
SPIRIT: Standard Protocol Items: Recommendations for Interventional Trials
SSC: Surviving Sepsis Campaign
