# Supplementary material for: Restrictive Fluid Administration vs. Standard of Care in Emergency Department Sepsis Patients (REFACED Sepsis)—protocol for a multicenter, randomized, clinical, proof-of-concept trial
Source: Pilot Feasibility Stud. 2022 Mar 29;8:75. doi: 10.1186/s40814-022-01034-y (PMC8962933; doi:10.1186/s40814-022-01034-y)

Restrictive Fluid Administration vs. Standard of Care in Emergency Department Sepsis Patients - a Multicenter, Randomized Clinical Feasibility Trial

Acronym: REFACED Sepsis

TRIAL PROTOCOL

Version 2.3

Dec 05, 2021

EudraCT number: 2021-000224-35

ClinicalTrials.gov number: NCT05076435

Committee on Health Research Ethics – Central Denmark Region: 1-10-72-163-21

Sponsor-investigator:

Marie Kristine Jessen, M.D.

Research Center for Emergency Medicine

Department of Clinical Medicine

Aarhus University and Aarhus University Hospital

Palle Juul-Jensens Boulevard 99, Bygning J, Plan 1

8200 Aarhus N, Denmark

Phone: +4525333286

Email: marie.jessen@rm.dk

Content

Preface 6

List of abbreviations 7

Overview 8

Trial flow chart 10

Steering committee 11

Trial sites 12

Amendments 13

1. BACKGROUND 15

1.1 Sepsis – the participant population 15

1.1.1 Incidence and mortality 15

1.1.2 Pathophysiology and definition 15

1.1.3 Treatments of sepsis 15

1.2 Fluid – the trial intervention 16

1.2.1 Use of fluids 16

1.2.3 Fluid restriction; pros and cons 16

1.2.4 Use in sepsis 17

1.3 Trials in fluid and sepsis 17

2. TRIAL OBJECTIVES AND HYPOTHESES 19

3. TRIAL DESIGN 20

3.1 Overview 20

3.2 Allocation 20

3.3 Interventions 20

3.3.1 Restrictive fluid administration 20

3.3.2 Standard care 22

3.3.3 Co-interventions 22

3.3.4 Concomitant interventions 22

3.3.5 Criteria for modification of interventions for a given trial participant and protocol violations 22

3.3.5 Assessment of participant compliance 23

3.4 Blinding 23

3.5 Trial procedures 23

3.5.1 Patients 23

3.5.2 Clinical personnel 23

4. SETTING AND PATIENT POPULATION 24

4.1 Setting 24
4.2 Inclusion criteria 24
4.3 Exclusion criteria 24
4.4 Co-enrollment 25
5. OUTCOMES 25
5.1 Primary outcome 25
5.1.1 Definition 25
5.1.2 Feasibility aim 25
5.2 Secondary outcomes 25
5.2.1 Definitions 25
5.2.2 Elaboration on secondary outcomes 26
5.4 Safety and harm 26
5.4.1 General consideration 26
5.4.2 Definitions for adverse events and reactions 27
5.4.3 Assessment of adverse events 28
5.4.4 Definitions of serious adverse reactions in REFACED Sepsis 28
5.4.5 Definitions of Suspected Unexpected Serious Adverse Reaction (SUSAR) in REFACED Sepsis 29
6. SAMPLE SIZE CALCULATION AND STATISTICAL ANALYSIS PLAN 30
6.1 Sample size calculation 30
6.2 Statistical analysis plan 30
6.2.1 General considerations 30
6.2.2 Primary and secondary outcomes 31
6.2.3 Missing data 31
6.2.4 Statistical stopping criteria 31
7. DATA COLLECTION AND MANAGEMENT 31
7.1 Data collection process 31
7.1.2 Variables 32
7.1.3 Pre-sepsis baseline characteristics 33
7.1.4 Sepsis characteristics 34
7.1.5 Variables obtained at randomization (the closest measured around time for randomization): 34
7.1.6 Variables obtained at 6-, 12- and 24 hours after randomization: 34
7.1.7 Variables on every day until 7 days from enrollment 35
7.1.8 Variables obtained at day 30 and day 90 after randomization: 36
7.2 Data quality and validity 36

7.3 Data storage and security 36
7.4 Data access 37
8. CLINICAL TREATMENT 37
8.1 Screening and enrollment 37
8.1.2 Variables 37
9. ETHICAL CONSIDERATIONS 38
9.1 Clinical equipoise 38
9.2 Research in sepsis 39
9.2.1 General considerations 39
9.2.2 Danish regulations 40
9.2.3 Regulations in relation to the current trial for patients physically or mentally incapable of giving consent 40
9.3 Procedures 41
9.3.1 Ethical review committee 41
9.3.2 Trial-specific procedures 41
9.3.3 Insurance 43
10. MONITORING 43
10.1 Good Clinical Practice monitoring 43
11. TIMELINE AND ENROLLMENT 44
11.1 Timeline 44
11.2 Feasibility 45
11.3 Enrollment 45
12. PUBLICATION PLAN 45
13. DATA SHARING 46
14. FUNDING 46
15. TASKS AND RESPONSIBILITIES 47
References 48
Appendices 52
Appendix 1: Conflict of interest disclosures for the steering committee members 52
Appendix 2: Sequential organ failure assessment (SOFA) score 54
Appendix 3: Procedure for reporting serious adverse events and serious adverse reactions from randomization to day 7 in REFACED Sepsis 55
Appendix 4: Product summaries of used fluids 56
Appendix 5: Informed consent forms 57

Appendix 6: Good-clinical-practice monitoring plan 61
Appendix 7: Draft of CONSORT flow diagram 62

Preface

The "Restrictive Fluid Administration vs. Standard of Care in Emergency Department Sepsis Patients - a Multicenter, Randomized Clinical Feasibility Trial" (REFACED Sepsis) will be conducted according to this protocol. The trial will be conducted in accordance with all applicable national and international laws, regulations, and guidelines including the revised version of the Declaration of Helsinki[1], European regulations[2], and the international Good Clinical Practice guidelines[3]. The trial and this protocol are developed in accordance with the International Conference on Harmonization (ICH) guidelines[3-5] and the Standard Protocol Items: Recommendations for Interventional Trials (SPIRIT) statement[6,7]. The principal investigator wrote the protocol with input from the steering committee. Any substantial changes or amendments to the protocol will be clearly documented and communicated to all relevant parties.

05-12-2021

Marie Kristine Jessen, M.D. Date

List of abbreviations

ED: Emergency department

eCRF: electronic case report form

Fi02: Fraction of inspired oxygen

GCP: Good Clinical Practice

ICU: Intensive care unit

ITT: Intention-to-treat

IV: Intravenous

LOS: Length of stay

MAP: Mean arterial pressure

Pa02: Partial pressure of oxygen

RRT: Renal replacement therapy

SAE: Serious adverse event

SAR: Serious adverse reaction

SOFA: Sequential organ failure assessment

SPIRIT: Standard Protocol Items: Recommendations for Interventional Trials

SSC: Surviving Sepsis Campaign

SUSAR: Suspected unexpected serious adverse reaction

Overview

Registry and trial number	EudraCT number: 2021-000224-35, ClinicalTrials.gov number: NCT05076435
Date of registration	EudraCT: 2021-05-03, ClinicalTrials.gov: 2021-10-13
Sources of monetary or material support	Carl and Ellen Hertz foundation, Frimodt-Heineke Foundation, Ruth & Holger Hesses Memorial Fund, Health Research Foundation of Central Denmark Region, "Akutpuljen" Central Denmark Region, and Aarhus University
Sponsor-investigator	Marie K. Jessen on behalf of Aarhus University
Contact	Marie K. Jessen, marie.jessen@rm.dk
Title	Restrictive Fluid Administration vs. Standard of Care in Emergency Department Sepsis Patients - a Multicenter, Randomized Clinical Feasibility Trial (REFACED Sepsis)
Country of recruitment	Denmark
Condition studied	Sepsis
Interventions	Restrictive intravenous fluid administration
Comparator	Standard intravenous fluid administration
Inclusion criteria	All of the below criteria fulfilled:<br>1) Unplanned emergency department admission<br>2) Age ≥ 18 years<br>3) Sepsis defined as<br>a) suspected infection by the treating clinician AND<br>b) blood cultures drawn AND<br>c) IV antibiotics administered or planned AND<br>d) infection-related increase of SOFA-score ≥ 2<br>4) Expected hospital stay > 24 hours as deemed by treating clinician
Exclusion criteria	Any of the following:<br>1) ≥ 500 ml of fluids given prior to randomization<br>2) Invasively ventilated or vasopressors<br>3) Known or suspected severe bleeding<br>4) Known or suspected pregnancy<br>5) Prior enrollment in the trial<br>6) Patients, who the clinician expect not to survive the next 24-hours

Study type	Interventional<br>Intervention model: Parallel group	Allocation: Randomized (1:1)<br>Masking: None
Date of first screening	To come	
Target sample size	124	
Recruitment status	Planning	
Primary outcomes	24-hour intravenous crystalloid fluid administration	
Key secondary outcomes	Feasibility measures:<br>• Number of patients with major protocol violations,<br>• Number of patients screened vs included<br>• Time from admission to inclusion<br>• Number of patients lost to follow up in terms of 24-hour fluids<br>• Accumulated serious adverse reactions and events (SARs + SUSARs) within 48 hours in-hospital<br>Total fluids (oral and intravenous) at 24 hours,	

Trial flow chart

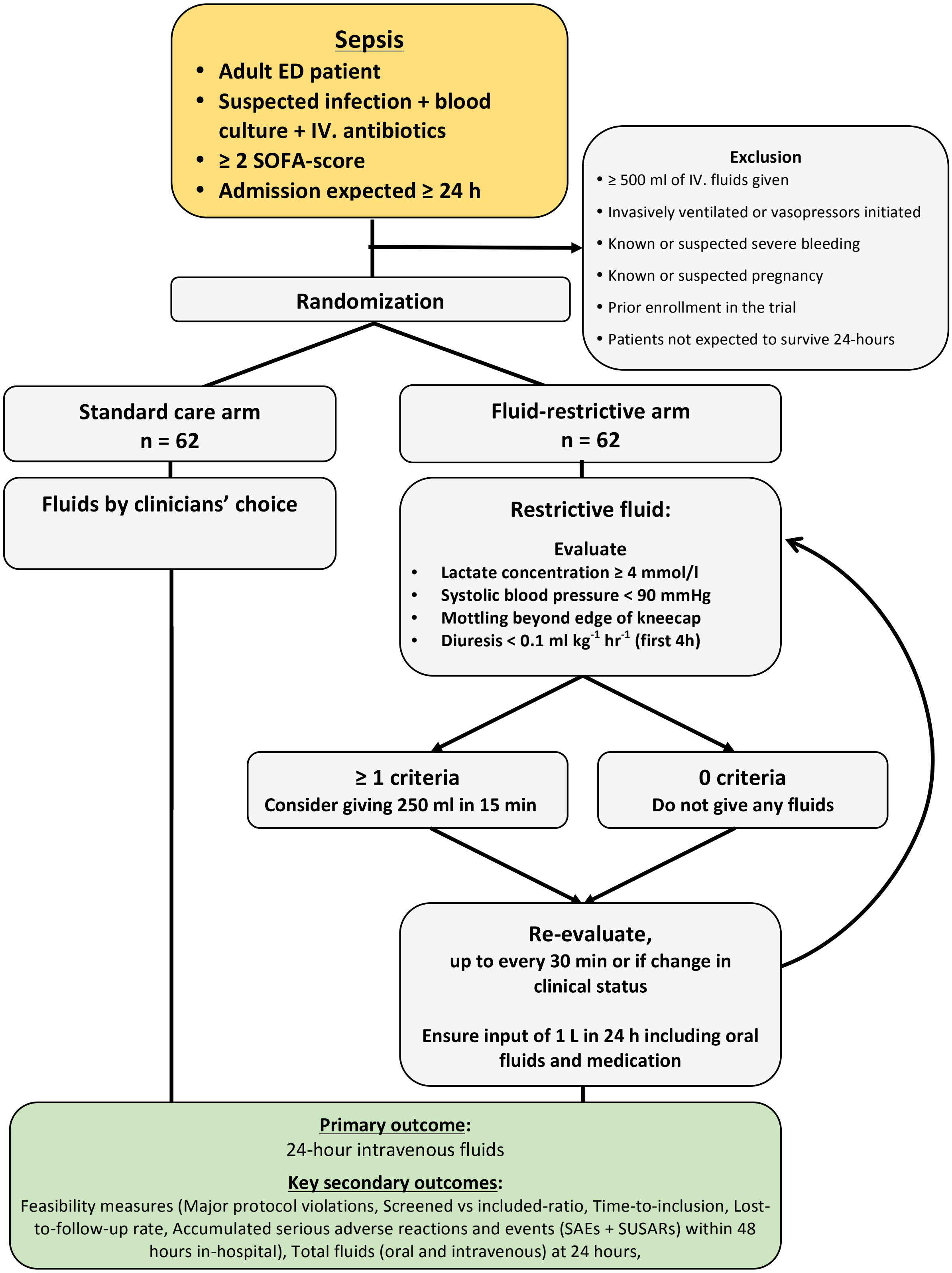

Steering committee

Marie K. Jessen, M.D.
Ph.D. fellow / Sponsor investigator
Research Center for Emergency Medicine
Department of Clinical Medicine
Aarhus University and Aarhus University Hospital

Lars W. Andersen, M.D., M.P.H., Ph.D., D.M.Sc.
Associate Professor
Research Center for Emergency Medicine
Department of Clinical Medicine
Aarhus University and Aarhus University Hospital

Hans Kirkegaard, M.D., Ph.D., D.M.Sc.
Director, Professor
Research Center for Emergency Medicine
Department of Clinical Medicine
Aarhus University and Aarhus University Hospital

Jens Aage Kølsen Petersen, M.D., Ph.D.
Associate Professor
Department of Anesthesiology
Department of Clinical Medicine
Aarhus University and Aarhus University Hospital

Anders Perner, M.D., Ph.D.
Professor
Department of Intensive Care,
Rigshospitalet and University of Copenhagen

Ranva Espegård Hassel, MD, Consultant physician,
Site investigator
Department of Emergency Medicine
Aarhus University Hospital
Palle Juul-Jensens Boulevard 161, Bygning J, Plan 2
8200 Aarhus N, Denmark
Telephone-number: +45-40514203
Email: ranva.hassel@rm.dk

Wazhma Hayeri, MD
Site investigator
Department of Emergency Medicine
Regional Hospital Randers
Skovlyvej 15
8930 Randers NØ, Denmark
Telephone-number: +45-60621985
Email: wazhay@rm.dk

Peter Kristensen, MD
Site investigator
Department of Emergency Medicine
Regional Hospital Viborg
Heibergs Alle 5A
8800 Viborg, Denmark
Telephone-number: +45-61360304
Email: petkre@rm.dk

Conflicts of interest

The members of the steering committee have no conflicts of interest related to the current trial. A list of all conflict of interests is provided in Appendix 1.

Trial sites

Aarhus University Hospital

Palle Juul-Jensens Boulevard 99

8200 Aarhus N

Denmark

Regional Hospital Randers

Skovlyvej 15

8930 Randers NØ

Denmark

Regional Hospital Viborg

Hospitalsenheden Midt

Heibergs Alle 5A

8800 Viborg

Denmark

Amendments

Version 1.0 to 1.1

- Clarification of data collection of pre-enrollment data (section 7.1.2)
- Clarification of screening and enrollment process (section 8.1)
- Clarification of consent and access to data from authorities (section 9.3.2)
- Clarification of publication of results regardless of outcome (section 12)

Version 1.1 to 1.2

- Safety and harm: Addition of section describing timing and classification of adverse events (section 5.4.3) and reporting (section 5.4.6) + appendix 3 (new version)
- Clarification that the precise IMP has to be noted on paper-CRF (section 7.1)
- Clarification of uploading process to EudraCT as soon as possible (section 12)

Version 1.2 to 1.3

- Clarification of end-of-trial report and notice to EudraCT (section 5.4.6)

Version 1.3 to 2.0

- Clarification of the acute medical trial and inclusion (section 9.2.3 and 9.3.2)
- Clarification of collection of data on screened patients (section 7.1.2)
- Clarification of consent procedures (section 9.3.2)

Version 2.0 to 2.1

- Clarification of collection of data on screened patients (section 7.1.2)

Version 2.1 to 2.2

- Addition of registration data from EudraCT and Clinicaltrials.gov
- Clarification of statistics (section 6.2.2)
- Clarification of screening population and successful inclusion (section 5.2.1 and 7.1.2)
- Clarification of collected data-variables i.e. comorbidities, timing of SOFA-score etc. (section 7.1.2+7.1.6 +7.1.9)

Version 2.2 to 2.3

- Correction of statistics in secondary outcomes (section 6.2)

1. BACKGROUND

1.1 Sepsis – the participant population

1.1.1 Incidence and mortality

Sepsis is very common in emergency department (ED) patients. Sepsis and suspected infection accounts for approximately 43% of all patient admissions in a Danish medical ED [8] and sepsis accounts for more than 500,000 yearly patient visits in the United States.[9] Patients presenting to the ED with suspected infection and sepsis are at risk of disease progression to septic shock (7-26%[10-13]) and ultimately death with mortality ranging from 10 to 23%.[14-16] It has been estimated that 15% of all deaths in Denmark are caused by sepsis.[17] In addition, the morbidity and effect on health-related quality of life after sepsis is significant.[18-20]

1.1.2 Pathophysiology and definition

Sepsis is defined as life-threatening organ-dysfunction caused by a dysregulated host-response to infection.[21] Sepsis is not a specific illness but rather a syndrome encompassing a still-uncertain pathobiology.[21] It can be identified by a constellation of clinical signs and symptoms in a patient with suspected infection caused by an entering bacteria, vira or fungi activating the immune system. Sepsis is a heterogeneous disease with a variety of sources of infection causing variable degrees of organ dysfunctions. The most frequent sources of infection are pneumonia, abdominal, urinary tract, and soft tissue infections.[8,22,23]

Sepsis is defined in accordance with the SOFA-score.[21] SOFA-score reflects organ dysfunction in the following organs/systems: respiratory, coagulation, liver, cardiovascular, central nervous system, and renal (see appendix 2). Sepsis is present if the patient has a suspected or verified infection and an increase in SOFA-score ≥ 2 from baseline caused by or suspected to be caused by the infection.

1.1.3 Treatments of sepsis

The mainstay of sepsis treatment in the initial phase includes intravenous (IV) antibiotics and fluids, source control, and supportive care, if necessary.[24]

The international Surviving Sepsis Campaign (SSC), which is supported by 25 medical societies, guides in treatment of septic shock, but does not give any recommendations on sepsis patients not in overt shock in general, despite the fact that sepsis is almost 60 times as common as septic shock.[8] The 1-hour bundle, introduced by SSC, recommends initiation of sepsis-treatment including fluids for hypotension/lactate > 4mmol/L within 1 hour [25]. In general, many sepsis studies have been conducted – especially in septic shock – but only very few have been proved effective leaving the clinician with only few evidence-based interventions for treatment.

1.2 Fluid – the trial intervention

1.2.1 Use of fluids

Intravenous fluid therapy is one of the most common therapeutic interventions performed in the ED. The most frequently used fluid, normal saline (0.9% NaCl), was described more than 100 years ago and the use was introduced to cholera patients.[26] Other crystalloids and colloids have been introduced, especially with the intention to balance solutions by electrolyte composition and osmolality to approximate to human plasma.

IV fluids for patients are used for different reasons; resuscitation (i.e., mostly to raise blood pressure and tissue perfusion), replacement of loss (e.g., vomit, diarrhea, perspiration, dehydration), maintenance to cover daily needs, correction of electrolytes, and as adjuvant for medications given intravenously.

The goals of fluids administration in terms of resuscitation is to increase intravascular volume.[27] The increased intravascular volume is intended to improve cardiac output and tissue oxygenation/perfusion.[24] The requirements for and response to fluid resuscitation vary greatly during the course of any critical illness. No single physiological or biochemical measurement adequately reflects the complexity of fluid depletion nor the response to fluid resuscitation in acute illness.[27]

Although the physiology of fluid treatment is not fully understood and elucidated, it probably should be regarded as a kind of medication with both intended effects and adverse reactions.[27] However, evidence to decide on this is sparse; "What is the optimal fluid treatment?" remains an unanswered question.

1.2.3 Fluid restriction; pros and cons

Recent fluid resuscitation and use seems to be quite liberal, in accordance with SSC, used for the above-mentioned reasons (section 1.2.1). However, too liberal fluid resuscitation may potentially result in worsening of tissue edema (including lungs), challenge the heart and lengthen the duration of mechanical ventilation. Liberal fluid resuscitation may also result in increased hemodilution, which may induce use of concomitant interventions (e.g., transfusion of blood products). Intravenous fluid administration has also been suggested to cause destruction of the capillary wall, causing further tissue edema. Restrictive fluid therapy may be beneficial in reducing venous backpressure and organ edema, thereby improving function of organs such as the lungs, gut, and kidneys.[28,29] On the other hand, fluid restriction may compromise peripheral and/or organ perfusion through reduced cardiac output and thereby reduced microcirculation from the arterial side.[30]

1.2.4 Use in sepsis

Fluid resuscitation has, as mentioned, been a corner stone of sepsis treatment for years. Liberal fluid therapy for septic patients has been recommended since the first Surviving Sepsis Campaign guideline (SSC, 2003).[31] The SSC's 2016-guidelines recommend at least 30 mL/kg of IV crystalloid fluid to be given within the first 3 hours (strong recommendation, low quality of evidence) for patients in septic shock.[24]

However, as seen, fluid resuscitation is guided by low quality of evidence[24] and studies show great variation in practice.[16,32,33] A yet unpublished study performed in our research group showed a great variability in fluids for sepsis patients with a range from 200-15870 ml. Patients with sepsis received approximately 3762 ml (standard deviation: 1839) fluids in total for 24 hours and patients with sepsis, blood cultures drawn and intravenous antibiotics received 2670 ml intravenous fluids (SD 1695). The physiology of fluid resuscitation in sepsis is not fully understood but fluid overload has been suggested to be harmful in multiple studies. [16,34-43] Multiple studies have, reproducibly, demonstrated that only approximately 50% of hemodynamically unstable patients will respond to a fluid challenge [16,44] – so called fluid responders. And it is unclear if the fluid responders obtain benefits from fluid beyond those observed as short-term hemodynamic effects.

1.3 Trials in fluid and sepsis

Within the last decade, there has been an increase in observational studies and interventional trials on fluid and septic shock with a number of randomized trials ongoing.[33,35,45,46]

A systematic review of predominantly observational studies found that positive fluid balance was associated with increased mortality.[35] A recent review and meta-analysis of randomized trials found no statistically significant difference between lower vs. higher fluid volumes in all-cause mortality. The study only included five trials and found low quality of evidence supporting the decision on the volumes of IV fluid therapy in adults with septic shock and sepsis. All five trials were in the intensive care units (ICU) setting.[47]

A recent randomized pilot trial by Hjortrup et al. (included in the above mentioned review) was able to reduce volumes of resuscitation fluids with a restrictive fluid protocol in septic shock patients in the ICU.[45] Another pilot trial in the ED by Macdonald et al. was able to reduce fluids with 30% with a fluid restrictive and early vasopressor approach in patients with sepsis associated hypotension.[46] The patient-centered outcomes in the study by Hjortrup et al. pointed towards benefit with fluid restriction with no negative effects on hemodynamic measurements. However, the trial was underpowered to show differences in clinical outcomes.[45,48] Both studies are now enrolling patients in large-scale randomized trials; the CLASSIC-trial (ClinicalTrials.gov Identifier: NCT03668236) enrolling ICU patients in Europe and the REFRESH-

trial/ARISE-FLUIDS (ClinicalTrials.gov Identifier: NCT04569942) enrolling ED patients in Australia and New Zealand. Another randomized trial, CLOVERS (ClinicalTrials.gov Identifier: NCT03434028), is enrolling persistent hypotensive patients in EDs in United States to either liberal or restrictive fluids for 24 hours.[49]

Although trials are currently exploring fluid strategies in patients with hypotension and septic shock [45,50], there are no studies on fluid administration in patients with early sepsis without shock/hypotension. Research within this field has been requested by experts.[26,51] As fluid administration is very frequent, but not evidence-based, and carries potential risks, we consider it to be of great interest for society and patients to perform research in this area. Fluid resuscitation is a key intervention in sepsis, but the optimal amount of fluid to be given has not been established. The present trial is a feasibility trial, i.e., a trial assessing the feasibility of the proposed protocol in a clinical setting. The aim is to investigate the ability to reduce fluid volume using the given protocol. Should the trial prove feasible with separation between the two interventions, a large-scale trial assessing patient important outcomes is intended.

2. TRIAL OBJECTIVES AND HYPOTHESES

Primary objective: To test if an IV fluid restrictive protocol in ED patients with sepsis is feasible, i.e., if the protocol decreases the IV fluid volumes administered.

Hypothesis: An IV fluid restrictive protocol is feasible in sepsis patients resulting in significantly less IV fluid volume administration as compared with standard care.

3. TRIAL DESIGN

3.1 Overview

This is an investigator-initiated, multicenter, randomized, parallel-group, open-labeled, feasibility trial investigating volumes of fluid within 24 hours in 124 patients with sepsis allocated to two different IV fluid regimens enrolled at three emergency departments in Central Region Denmark. The objective is to assess the feasibility of a trial comparing two approaches to intravenous fluid resuscitation of sepsis; a restrictive approach using four criteria vs. standard care. The hypothesis is that the restrictive approach is feasible and will result in less IV fluid given. The primary outcome is total intravenous fluid volume within 24 hours and key secondary outcomes include protocol violations, total fluids (intravenous and oral) within 24 hours, progression to septic shock, and SAEs/SUSARs.

3.2 Allocation

Patients fulfilling all inclusion criteria and no exclusion criteria will be randomized in a 1:1 ratio in blocks of varying concealed sizes stratified by site [52] at enrollment to one of two fluid treatment arms lasting for the first 24 hours of admission or until the patient is discharged within 24 hours. Randomization will be performed via the web-based randomization system provided in REDCap ensuring allocation concealment.

3.3 Interventions

3.3.1 Restrictive fluid administration

No IV fluids should be given unless one of the below mentioned occurs; in these cases, IV fluid may be given in measured amounts:

A fluid bolus of 250 ml (half of a 500 ml fluid bag) isotonic crystalloid may be given within 15 minutes if one of the following occurs (hypoperfusion criteria):

- Lactate concentration ≥ 4 mmol/l (arterial or venous blood gas/blood sample)
- Hypotension (systolic BP < 90 mmHg)
- Mottling beyond edge of kneecap (i.e., Mottling score >2)[53]
- Severe oliguria, i.e., diuresis < 0.1 ml/kg/h, during the first 4 hours of admission

The effect of a fluid bolus may be assessed after 30 minutes by re-evaluation of the four hypoperfusion criteria mentioned above by the treating clinician. If one or more of the criteria are still fulfilled, a fluid bolus as defined above may be repeated. At any time, the clinician can start vasopressors if deemed necessary.

Only isotonic crystalloids are to be given as resuscitation fluid; the type of isotonic crystalloid is free of choice. Crystalloid boluses are to be given via IV drip. Intravenous fluids may be given as carrier for medication, but the volume should be reduced to the lowest possible volume for the given medication.

In case of documented overt fluid losses (e.g., vomiting, large aspirates, diarrhea, drain losses, or ascites drainage or due to dehydration) IV fluid may be given to correct for the loss.

In case the oral/enteral route for water or electrolyte solutions is contraindicated or has failed as judged by the clinical team, IV fluids may be given to:

- Correct significant electrolyte deficiencies
- Ensure a total fluid input of 1 L per 24 h (fluids with medications and nutrition count as input).

If a patient undergoes surgery during the 24 hours inclusion period, they are temporarily out of the protocol, but we will encourage continuing restrictive fluid therapy.

The cut-off value of lactate was chosen based on SSC guidelines[24] and their one-hour bundle[54] and data indicating that the marked increase in mortality occur at lactate values above 4 mmol/l.[55,56] The mottling trigger is based on mottling score ≥ 2 (see figure below) as described by Ait-Oufella et al [53] and validated in a pre-hospital setting.[57] Severe oliguria is defined as urine output ≤ 0.1 ml/kg/hour and the criteria is only to be used within the first 4 hours of admission.

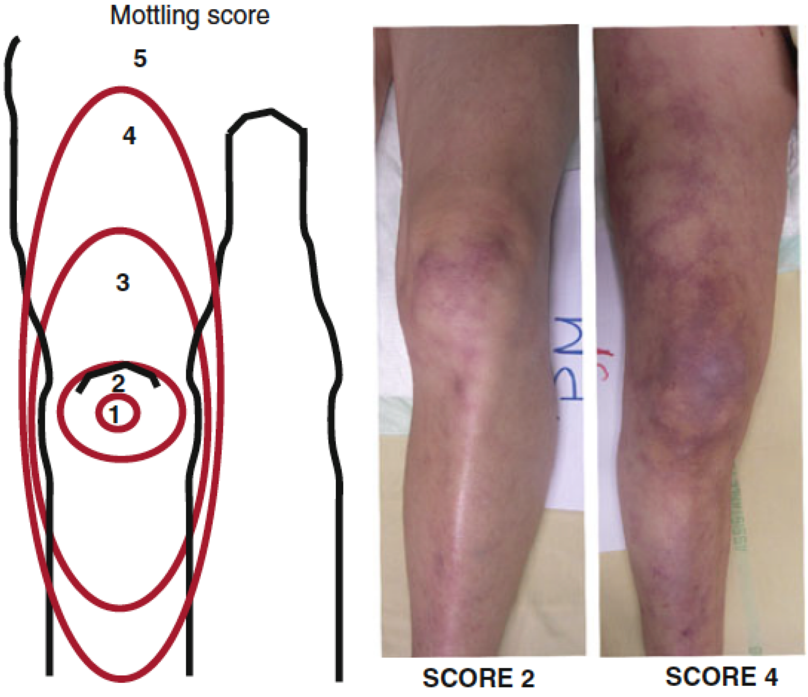

Figure showing Mottling score, copy with permission from Ait-Oufella et al.[53]

3.3.2 Standard care

Fluid may be given intravenously in amounts of the clinician's choice as usual. There will be no upper limit for the use of either IV or oral/enteral fluids. In particular:

- IV fluids should be given in the case of hypoperfusion or circulatory impairment and should be continued as long as hemodynamic variables improve as chosen by the clinicians. These criteria are based on the SSC guideline[24]
- IV fluids should be given as maintenance if the departments have a protocol recommending maintenance fluid
- IV fluids should be given to substitute expected or observed loss, dehydration or electrolyte derangements

3.3.3 Co-interventions

Types of fluids in both intervention groups:

- Fluids used for electrolyte disturbances: Fluids should be chosen to substitute the specific deficiency
- Fluids given to substitute overt loss: Isotonic crystalloids are to be used. If large amounts of ascites are tapped, then human albumin may be used.
- Blood products are only to be used on specific indications including severe bleeding, severe anaemia and prophylactic in case of severe coagulopathy.

3.3.4 Concomitant interventions

Patients should, besides the above-mentioned randomized treatments, be cared for as usual in terms of their infection. Treatment of sepsis is complex with multiple interventions and, as blinding of treating personnel is not feasible, use of several concomitant interventions may be influenced by allocated intervention arm. In order to minimize these potential differences, the use of concomitant interventions for sepsis should be based on the Danish Society of Infectious Disease guidelines and the one-hour bundle from SSC.

3.3.5 Criteria for modification of interventions for a given trial participant and protocol violations

The clinical team may at any time violate the protocol if they find it to be in the interest of the participant. We will have a 24-hour per day trial hotline to enable discussion between the clinicians caring for trial participants and the REFACED Sepsis trial team regarding protocol related issues.

Protocol violations are defined as follows:

Intervention group	Major protocol violation	Documentation
Restrictive fluid administration	IV fluids given if none of the bellow is true:<br>• 1 or more hypoperfusion criteria fulfilled<br>• To correct documented fluid loss<br>• To correct significant electrolyte deficiencies<br>• Fluid administered as carrier for medication (e.g. antibiotics)<br>• Ensure a total fluid input of 1 L per 24 h	Reasons for the protocol violation
Standard care	No IV fluids given	Reasons for the protocol violation

3.3.5 Assessment of participant compliance

We will monitor protocol compliance at the trial site through the electronic case report form (eCRF) in REDCap and alert sites in the case of clear violation (central monitoring). In addition, the trial will be monitored according to the Good Clinical Practice (GCP) directive and a monitoring plan conducted in collaboration with the GCP-unit at Aarhus and Aalborg Universities.

3.4 Blinding

Fluid restriction vs. standard care fluid therapy cannot be blinded for investigators, clinical staff, or participants.

3.5 Trial procedures

3.5.1 Patients

The trial procedures will be limited to the interventions given in the first 24 hours of admission. Data will be obtained from the study specific case report form and the electronic medical records.

3.5.2 Clinical personnel

Prior to the beginning of patient enrollment and continuously throughout the enrollment period, the clinical teams involved in the treatment of sepsis patients at the participating hospitals will be informed about the

trial. Clinical personnel will be informed about the background and objectives of the trial, the inclusion/exclusion criteria, the interventions, and the trial procedures they are involved in. We anticipate formal, in-person didactics continuously with informal sessions and emails as applicable in between as well as an educational video, especially due to the current COVID-19-pandemic.

4. SETTING AND PATIENT POPULATION

4.1 Setting

The trial will be conducted at three EDs at Aarhus University Hospital and Regional Hospital Randers and Viborg, Denmark. All three participating sites have clinical experience and expertise in treating sepsis patients.

4.2 Inclusion criteria

The research team, clinically working nurses and ED physicians will screen ED patients for the following inclusion criteria:

1) Unplanned emergency department admission
2) Age ≥ 18 years
3) Sepsis defined as
    a) suspected infection by the treating clinician AND
    b) blood cultures drawn AND
    c) IV antibiotics administered or planned AND
    d) An infection related increase of SOFA-score ≥ 2 from baseline[21]
4) Expected hospital stay > 24 hours as deemed by treating clinician

These broad inclusion criteria were chosen to investigate the effect of fluid administration in the entire, broad sepsis population and increase the likelihood of describing the entire 24 hours inclusion period. We will strive to enroll participants as soon as they fulfill the criteria in the ED. There is no exact maximum time frame for patient inclusion, however with the exclusion criteria of > 500 ml. IV fluids administered, the inclusion is expected and intended to happen within the first couple of hours.

4.3 Exclusion criteria

We will exclude patients fulfilling any of following exclusion criteria:

1) ≥ 500 ml of fluids given prior to randomization

2) Invasively ventilated or vasopressors initiated at the time of screening

3) Known or suspected severe bleeding judged by the treating clinician

4) Known or suspected pregnancy (women aged <45 years will have a pregnancy test performed before enrollment)

5) Prior enrollment in the trial

6) Patients, who the clinician expect not to survive the next 24-hours

4.4 Co-enrollment

There will be no general restrictions on entry into other clinical trials although this will be evaluated on a case-by-case basis.[58] However, patients enrolled in REFACED Sepsis will not be able to be enrolled in the CLASSIC trial (ClinicalTrials.gov identifier NCT03668236) if transferred to the ICU, since it would be contradictory to receive restrictive care in one of the studies and standard care in the other at the same time.

5. OUTCOMES

5.1 Primary outcome

5.1.1 Definition

The primary outcome will be the total amount of all administered intravenous, crystalloid fluids within 24 hours of randomization.

5.1.2 Feasibility aim

The primary result is to see, if patients in the restrictive arm have significantly less intravenous fluid administered within 24 hours than the standard-care group. This study is a feasibility study, and a future large-scale study is planned.

5.2 Secondary outcomes

5.2.1 Definitions

The secondary outcomes will be

- Feasibility measures:
    - Number of patients with major protocol violations,

- Number of patients screened positive (ie.with all inclusion criteria fulfilled and no exclusion criteria fulfilled) vs randomized
    - Time from admission to inclusion
    - Number of patients lost to follow up in terms of 24-hour fluids (e.g. due to discharge within 24 hours)
    - Accumulated serious adverse reactions and events (SAEs + SARs+ SUSARs) within 7 days in-hospital
- Total fluids (oral and intravenous) at 24 hours

We will also report in-hospital mortality and 30- and 90-day mortality (possible outcomes for the final large scale trial), in-hospital length of stay (LOS), mechanical ventilation within 7 days of admission (yes/no), vasopressor use within 7 days of admission (yes/no), and development or worsening of acute kidney failure according to the KDIGO3-criteria within 7 days of admission (also an SAE, see above) [59].

5.2.2 Elaboration on secondary outcomes

The study is a feasibility trial only powered to answer the primary aim. However, we regard the above mentioned as important feasibility measures. The trial will include additional outcomes focused on hemodynamics, organ failure, and long-term outcomes in order to be able to power calculate for the planned large-scale trial.

Both 30-day and 90-day survival will be obtained from electronic medical records or the Danish Civil Personal Register which allows for accurate and virtually complete follow-up.[60]

We will include 90-day survival as a measure of long-term survival. 90 days were chosen since it is unlikely that mortality later than that will be directly linked to the sepsis event or the trial interventions.

A vasopressor will be defined as any continuous infusion of noradrenaline, dopamine, dobutamine, terlipressin, vasopressin, phenylephrine, and/or adrenaline. Receiving vasopressors for a total of at least 3 hours on a given day is defined as receiving vasopressors for that day. Invasive ventilation is defined as mechanical ventilation through an endotracheal or tracheostomy tube, not only used for surgery for a total of at least 3 hours on a given day.

5.4 Safety and harm

5.4.1 General consideration

The trial will only be conducted in departments that are used to care for and treat sepsis patients. We therefore in general believe it is safe for individual patients to be enrolled into the REFACED Sepsis trial.

As described above, randomized trial data and data from observational and physiological studies do not provide firm evidence, that one of the interventions in the REFACED Sepsis trial is superior to the other. We therefore believe, that the REFACED Sepsis trial is safe for the patients also at the group level.

Generally, fluid administration is considered safe and is very commonly used in clinical practice. The overall benefit and potential harm will be captured in our secondary outcomes, and the clinical team will document any specific adverse events suspected to be related to the intervention.

The registration of the batch numbers and the expiry dates of the IV fluids and the identity of the clinician administering the fluid will be registered as per standard practice at the sites. These data will not be registered in the trial documents, but can be obtained by the Sponsor or the authorities if needed. We believe that this is a safe procedure because the IV fluids used in the REFACED Sepsis trial has been in clinical use for many years and the safety of single doses cannot be questioned. The same procedure was approved by the Danish Medicines Agency in the CLASSIC pilot trial (EudraCT no. 2014-000902-37) and the large-scaled CLASSIC trial (EudraCT no. 2018-000404-42).

The intervention arm in the present trial does not dictate which type of isotonic crystalloid that is to be used. SARs are defined from the Danish summaries of product characteristics of the most frequently used isotonic crystalloids in Denmark (normal saline (0.9% NaCl), Ringer-lactate, and Ringer-acetate). Participants in both intervention arms are expected to receive fluid resuscitation, but the amount is hypothesized to differ. The use of fluids has some obvious reactions presented in the Danish Summary Product Characteristics for the crystalloid solutions (see appendix 3+4).

5.4.2 Definitions for adverse events and reactions

The following definitions will be used[2]:

<u>Adverse event (AE)</u>: any untoward medical occurrence in a subject to whom a medicinal product is administered and which does not necessarily have a causal relationship with this treatment

<u>Adverse reaction (AR)</u>: All untoward and unintended responses to an investigational medicinal product related to any dose administered

<u>Serious adverse event (SAE)</u>: Any adverse event that results in death, is life-threatening, requires hospitalization or prolongation of existing hospitalization, results in persistent or significant disability or incapacity, or is a congenital anomaly or birth defect.

Serious adverse reaction (SAR): Any adverse reaction that results in death, is life-threatening, requires hospitalization or prolongation of existing hospitalization, results in persistent or significant disability or incapacity, or is a congenital anomaly or birth defect. The SARs are identified in the Danish Summary of Products Characteristics (SmPC) for the used crystalloids.

Suspected unexpected serious adverse reaction (SUSAR): a serious adverse reaction, the nature, severity or outcome of which is not consistent with the reference safety information (SmPC).

5.4.3 Assessment of adverse events

Timing

In all participants, we will assess the occurrence of SARs in the 7 days following randomization (the intervention period is 24 hours; 7 days allow for another 6 days of assessment after the intervention, which is clinically relevant in critically ill patients.

Classification of an event

We will make no inferences about a causal relationship between the intervention and the SARs but register the occurrence in the two groups and report them in the final report according to the definition given above. The investigators will classify SAEs (as per the definition above occurring within 7 days from randomization) and report them to the sponsor. If such a SAE is deemed both unexpected and related to the intervention by the investigator, it will be considered a SUSAR and reported as such. If the sponsor does not adjudicate the SAE as related to the intervention, this will also be noted in the final report to the Medicines Authorities.

5.4.4 Definitions of serious adverse reactions in REFACED Sepsis

Defined serious adverse reactions (SARs) are:

- General tonic-clonic seizures: Stiffening and/or jerking movements of all 4 extremities in a patient who becomes or is unconscious after randomization
- Anafylactic reactions defined as urticarial skin reaction AND at least one of the following observed after randomization:
    - Worsened circulation (>20% decrease in systolic blood pressure or >20% increase in vasopressor dose)
    - Increased airway resistance (>20% increase in peak pressure on the ventilator) if intubated

    - Clinical stridor or bronchospasm
    - Subsequent treatment with bronchodilators
- Central pontine myelinolysis seen on CT or MRI scan within the 7-day after randomization
- Hypernatremia defined as P-Na> 159 mmol/L on any plasma sample including point-of-care testing after randomization if hypernatremia is not present at randomization or worsening of a preexcisting hypernatriemia with an increase in P-Na of > 5mml/L from baseline (at enrollment)
- Severe hyperchloremic acidosis defined as pH< 7.15 AND p-chloride > 115 mmol/L on any plasma sample, including point-of-care testing after randomization if hyperchloremic acidosis is not present at randomization or worsening of a preexcisting hyperchloremia with an increase in p-chloride > 5 mml/L or decrease in pH>0.05 (AND pH<7.15 at baseline) from baseline (at enrollment)
- Severe metabolic alkalosis defined as pH>7.59 AND SBE>9mmol/L on any plasma sample, including point-of-care testing after randomization if metabolic alkalosis is not present at randomization or worsening of a preexcisting metabolic alkalosis with a increase in pH>0.05 and increase in SBE>5mml/L from baseline (at enrollment).

5.4.5 Definitions of Suspected Unexpected Serious Adverse Reaction (SUSAR) in REFACED Sepsis

SUSARs will be defined as serious reactions not described in the Summaries of Product Characteristics for the used crystalloids happening within 7 days of admission if not otherwise stated. Suspected Unexpected Serious Adverse Reactions (SUSAR) will be reported to the regulatory authorities as applicable.

5.4.6 Reporting

Any SAE adjudicated to be unexpected or related to the trial intervention by the investigator, will be reported within 24 hours to the sponsor or his delegate. If deemed a SUSAR by the sponsor, he will report it to the Danish Medicine Agency, the Ethics Committee and all trial sites within 7 days. No later than 8 days after the reporting, the Sponsor will inform the Danish Medicines Agency of relevant information on the Sponsor's and the investigator's follow-up action to the life-threatening or fatal SUSAR. Any other SUSARs will be reported to the Danish Medicines Agency no later than 15 days from the time when the Sponsor is informed.

In Appendix 3, SAEs seen frequently in critically ill sepsis patients are listed. The listed SAEs do not have to be reported to the sponsor within 24 hours of occurrence if judged not to be related to the intervention and

expected in the patent population in accordance with the flowchart. All SAEs not listed in Appendix 3 will be reported to sponsor within 24 hours of occurrence.

Once a year, the sponsor will submit a list of all SARs that have occurred at all sites during the trial period and a report on safety of the trial subjects to the Danish Medicines Agency and National Ethics Committee. The sponsor will notify and upload the results from the clinical trial including important adverse events on EudraCT using the "Declaration of the End of Trial Form" when the trial has been completed (no later than 90 days thereafter) and if earlier than planned, the reasons for stopping the trial.

6. SAMPLE SIZE CALCULATION AND STATISTICAL ANALYSIS PLAN

6.1 Sample size calculation

The trial will be powered to the primary outcome of 24-hour total intravenous fluids. Sample size calculation is based on unpublished data from an observational study conducted in three of the hospitals in Central Denmark Region. The study found that sepsis patients received a mean of 3762 ml (SD 1839) intravenous and oral fluid in 24 hours. Looking at only patients with sepsis, blood cultures drawn and intravenous antibiotics, patients received 2670 ml intravenous fluids (SD 1695). We conservatively estimate that the total amount of IV fluid in the control group will be approximately 2650 ml (standard deviation 1.7 L). We consider a mean difference of 1 L to be clinical meaningful and therefore estimate 1650 ml (standard deviation 1.7 L) in the intervention group. Based on these estimates, an alpha of 5%, a power of 90%, and a two-sample t-test, a sample size of 124 patients is required; 62 in each treatment arm. Using the same standard deviation of 1.7 L, which is even higher than the mean in the restrictive arm, is or most conservative estimate being able to segregate the two groups.

6.2 Statistical analysis plan

6.2.1 General considerations

The statistical reporting will adhere to the CONSORT guidelines.[61,62] All tests will be two-sided, a p-value < 0.05 will be considered significant, and all confidence intervals will have 95% coverage.

Patient inclusion and exclusion will be illustrated in a CONSORT flow diagram (see Appendix 7 for a draft).

We will include measures related to feasibility including the enrolled to screened ratio, time to randomization, and protocol adherence/major protocol violations. All analyses will be conducted in the intention-to-treat (ITT) population defined as all randomized participants for whom consent was obtained. We will perform the primary analyses adjusted for the stratification variable site.

The two groups will be compared in relation to baseline patient and sepsis characteristics using descriptive statistics.

6.2.2 Primary and secondary outcomes

To estimate the mean difference in fluid volume between groups, we will use linear regression with adjustment for the stratification variable. If the data is severely non-normally distributed, we will consider other appropriate options (e.g., "robust standard errors" or transformation). Other continuous variables will be analyzed similarly. For binary outcomes, we will use logistic regression adjusted for site and results will be presented as odds ratios.

6.2.3 Missing data

Missing data will be reported in the relevant publications. We do not expect any missing data for the primary outcome (except for those discharged within the 24 hours) or the key secondary outcomes. Patients discharged within 24 hours or who died within 24 hours, will be included in the ITT analysis with the amounts they received until discharge/death. We do not expect missing data on mortality or adverse events. Multiple imputation using known risk factors for outcomes in sepsis will be used to impute values for patients with missing data if missing data is substantial (> 10%).

6.2.4 Statistical stopping criteria

Since the primary outcome is not mortality, there will be no formal stopping criteria for efficacy. There will be no predefined stopping criteria for futility since enrollment of the full cohort might allow for detection of differences in other outcomes even if the primary outcome is negative.

7. DATA COLLECTION AND MANAGEMENT

7.1 Data collection process

Enrollment and randomization will be performed by the treating team directly in the trial specific REDCap-site. The treating team will register limited data in REDCap including patient identifier (i.e., Danish Central Personal Register number), site and inclusion/exclusion criteria, and limited trial specific data in the electronical medical record to notify the enrollment of the patient in the trial. This will include study ID, timing of enrollment and allocation. A paper-CRF (A bedside REFACED Sepsis resuscitation chart) will be placed at the patient’s bedside collecting data on fluid management filled out by the treating team during the 24 hours; timing of fluids administered, indication for fluid bolus, fluid type (the precise investigative

medicinal product (IMP); 1) Sodium Chloride solution 0.9% B.Braun, Sodium Chloride 9mg/ml Fresenius Kabi, Ringers) and volume and time to re-evaluation and protocol violations and reasons for these. Further data will be obtained from the electronic medical journal by the research team as described below including for example vital signs, blood tests etc.; all data will be based on measurements and assessments made by the clinical team. A trained member of the research team (either the sponsor, site investigators or employed research nurses and medical students) will be responsible for data collection and entry into the eCRF from the electronic medical journal and from the paper-CRF. Data will be entered directly into the database software from the electronic medical journal (see section 7.4). The paper-CRF and e-CRF in REDCap will both be developed and tested and validated before initiation of the trial at all sites to optimize the use and be able to uniform the data abstraction.

7.1.2 Variables

All sepsis patients at the participating sites will be entered into a screening log by daily going through all adult, admitted patients who had a blood culture perfomed (screening criteria) and check if these patients fulfilled inclusion criteria and exclusion criteria. The screening log will be carried out by a trained member of the research team (either the sponsor, site investigators or employed research nurses and medical students). For those not randomized, a specific reason for non-inclusion/exclusion will be documented. The screening log will for all screened patients include variables for inclusion, exclusion and for some patients the logistical reasons for not including the patients. This data will be collected either before informed consent by the “legal guardian” or for patients screened and excluded without informed consent. Data collected from the electronic patient journal before consent will be passed on to the primary investigator for use in the trial, as well as legal authorities by request. Screening for patients will be performed “real-time” by the trained research team and the treating teams, but the screening log will be filled out once daily by the trained research team going through all patients admitted since the last screening log was performed. All randomized patients will be entered into the main REDCap database.

A detailed data dictionary that clearly defines all included variables in the eCRF (elaboration of 7.1.3-7.1.8) will be created prior to patient enrollment. The data dictionary will provide the name of the variable (including the code used in the database), a detailed definition of the variable, categories for categorical variables, and units and ranges for continuous variables.

The number of collected variables will be kept relatively small to limit resource use and data entry mistakes. Below is provided a brief overview of the included variables but details are reserved for the data dictionary. The following variables will be obtained on all included patients and collected from the electronic patient journal or the paper-CRF (7.1.3-7.1.8):

7.1.3 Pre-sepsis baseline characteristics

Trial related variables

Study ID

Site

Inclusion criteria

Exclusion criteria

Date and time consent for data collection is obtained

Patient demographics and characteristics

Name

Unique patient identifier (CPR number)

Age

Sex

Height

Weight

Conditions/medications prior to enrollment

Co-morbidities:

- Previously admitted for:
    - Heart failure + ejection fraction and/or diastolic dysfunction
    - Myocardial infarction and/or cardiac arrest
    - Stroke
- Diagnosed with:
    - Asthma or chronic obstructive pulmonary disease Y/N,
    - Chronic treatment for arterial hypertension Y/N,
    - Chronic treatment for diabetes Y/N,
    - Haematological malignancy Y/N,
    - Metastatic cancer Y/N,
    - Dementia Y/N
    - Atrial fibrillation (chronic or paroxysmal)

- Habitual s-creatinine obtained from the laboratory systems in the electronic patient journal (worst within 5-90 days prior to admission)
- Clinical frailty index

7.1.4 Sepsis characteristics

Site of infection:

Pulmonary

Abdominal

Urinary tract

Soft tissue

Other/unknown

7.1.5 Variables obtained at randomization (the closest measured around time for randomization):

Values for SOFA-score at admission [21]

Results of blood samples (standard lab. Values + arterial/venous blood gas)

- Lowest values: haemoglobin, platelets, eGFR,
- Highest values: lactate, kreatinin, bilirubin, C-reactive protein, leukocytes (white blood count),

Vital parameters

- Highest heart rate, respiratory rate,
- Lowest values of mean arterial blood pressure
- Highest values of arterial or venous lactate concentration or from a blood sample

Antibiotics: Type, timing and dosage and administration form

Volume of resuscitation fluids (crystalloids, colloids and blood products in ml) until randomization

Other fluids including blood products, nutrition, fluid with medication

7.1.6 Variables obtained at 6-, 12- and 24 hours after randomization:

Hourly volumes of resuscitation fluid with specification of indication for each fluid bolus

Other fluids including blood products, nutrition, fluid with medication

Highest creatinine within 24 hours to evaluate KDIGO-criteria

Only 24hours: To evaluate worst SOFA-score within 24 hours:

Results of blood samples (standard lab. Values + arterial/venous blood gas)

- Lowest values: platelets, Pa02 (and Fi02)
- Highest values: lactate, bilirubin,

Use of renal replacement therapy Y/N

Protocol violations

Non-invasive ventilation Y/N

On mechanical invasive ventilation or vasopressor Y/N

Status: dead, Y/N

Only 24hours: SAEs (see section 5.4.3) + SARs (see section 5.4.4)

SAEs on this day (y/n for everyone)

- Cerebral ischemic event
- Myocardial ischemic event
- Intestinal ischemic event
- Limb ischemia
- New onset of severe acute kidney injury

SARs on this day (y/n for everyone)

- General tonic-clonic seizures
- Anaphylactic reactions
- Central pontine myelinolysis
- Severe hypernatremia
- Severe hyperchloraemic acidosis
- Severe metabolic alkalosis

Only 24 hours: SUSARs

7.1.7 Variables on every day until 7 days from enrollment

On mechanical invasive ventilation or vasopressor Y/N

SAEs (see section 5.4.3 + see above)

SUSARs (see section 5.4.5)

Status: dead, Y/N

7.1.8 Variables obtained at day 30 and day 90 after randomization:

Status: dead, Y/N

7.1.9 Other variables

Discharge dispositions: Home, home with assistance for personal care, nursing home, rehabilitation, other hospital facilities, other

7.2 Data quality and validity

We will train all clinically working nurses and physicians involved in the treatment of enrolled patients to optimize data quality and validity. This will further be optimized by having trained trial personnel entering all data from the paper-CRF and electronic medical journal according to a detailed data dictionary. Research Electronic Data Capture (REDCap) (see section 7.4) is designed such that data forms contain field-specific validation checks ensuring that mandatory fields are filled out and that continuous variables are within predefined ranges. The eCRF will be validated thoroughly before enrollment of the first patient.

7.3 Data storage and security

The database application we will use is REDCap.[63] REDCap is a professional database that provides a user-friendly interface. The REDCap data management system is secure, fully compliant with all regulatory guidelines, and includes a complete audit-trail for data entry validation. Through these mechanisms, as well as relevant training for all involved parties, patient confidentiality will be safeguarded. REDCap is available for free at participating sites.

The case report form and the consent form for each patient will initially be stored in a secure, locked place at the individual sites and will also be uploaded to REDCap. Every half-year the paper-CRFs will be transported to the Research Center for Emergency Medicine in Aarhus while a copy will remain at the sites. Here they will be securely stored in locked cabinets, where only the principal investigator and the research nurse will have access. The files will be stored for 15 years after the end of the trial, where after they will be destroyed, or else they will be stored according to new regulations if implemented prior to enrollment of the first patient.

Data will be handled according to all relevant Danish laws including the General Data Protection Regulation ("Databeskyttelsesforordningen") and the Data Protection Act ("Databeskyttelsesloven"). The project will be registered with the Central Denmark Region's internal list of research projects. De-identified data will be made publicly available 9 months after the publication of the outcome data according to the recent ICMJE recommendations. All trial-related documents will be public available at a trial-website

including those of the trial master file, the eCRF template, instructions, educational material etc. Patient related data will not be accessible through this website.

7.4 Data access

Each patient will receive a unique trial identification number. During the trial, the sponsor investigator and will have access to the entire database, while primary site investigators, research nurses and employed study personnel will have access to data from their own sites. All employes at enrolling sites with involvement in patient inclusion, enrollment and treatment, will be granted access to the REDCap database for their own site with a personal user name and a personal access code. The Good Clinical Practice unit, regulatory agencies, and other relevant entities will have direct access to patients' records and to all relevant trial data including the case report form as applicable.

8. CLINICAL TREATMENT

8.1 Screening and enrollment

Patients will be screened and randomized followed by enrollment by the treating clinical team or the trained study personnel. Screening for patients will be performed "real-time" by the trained research team and the treating teams looking through lists of patients arriving or admitted to the EDs with clinical symptoms of sepsis and fulfilling inclusion criteria. As soon as fulfillment of inclusion criteria are proven, the "first legal guardian" will be approached to get informed consent. This trial guardian has received required information (written and oral) about the trial to be able to make an informed decision about the patient's study participation and enrollment. The oral consent will be followed by a written consent within a few hours when the critical situation has calmed down (see 9.3.2).

The clinical management of included patients, other than fluid strategy according to randomization, will be at the complete discretion of the treating clinical team in order to test the interventions in a real-life clinical scenario. As described in 3.3.4 "Concomitant interventions" clinicians will be encouraged to follow national standards by the Danish Society of Infectious Disease.

8.1.2 Variables

All sepsis patients at the participating sites will be entered into a screening log by daily going through all admitted patients and check if these patients fulfilled criteria for sepsis and had intravenous antibiotics administered and a blood culture drawn. The screening log will be carried out by a trained member of the research team (either the sponsor, site investigators or employed research nurses and medical students). For

those not randomized, a specific reason for non-inclusion/exclusion will be documented. The screening log will for all screened patients include variables for identification (CPR-number), hospitalization date, inclusion (blood culture obtained yes/no, intravenous antibiotics administered yes/no, suspected infection in charts yes/no, ≥2 SOFA score (requiring results from laboratory values), exclusion (invasively ventilated yes/no, vasopressors given yes/no, severe bleeding yes/no, pregnancy yes/no, prior enrollment yes/no, not suspected to survive 24 hours yes/no) and for some patients the logistical reasons for not including the patients. This data will be collected either before informed consent by the "first trial guardian" or for patients screened and excluded without informed consent. Data will be used in the study to describe patient flow in the CONSORT diagram. Screening for patients will be performed "real-time" by the trained research team and the treating teams, but the screening log will be filled out once daily by the trained research team going through all patients admitted since the last screening log was performed. All randomized patients will be entered into the main REDCap database.

A detailed data dictionary that clearly defines all included variables in the eCRF (elaboration of 7.1.3-7.1.8) will be created prior to patient enrollment. The data dictionary will provide the name of the variable (including the code used in the database), a detailed definition of the variable, categories for categorical variables, and units and ranges for continuous variables.

The number of collected variables will be kept relatively small to limit resource use and data entry mistakes. Below is provided a brief overview of the included variables but details are reserved for the data dictionary. The following variables will be obtained on all included patients and collected from the electronic patient journal or the paper-CRF (7.1.3-7.1.8):

9. ETHICAL CONSIDERATIONS

9.1 Clinical equipoise

Fluid resuscitation is a key intervention in sepsis and septic shock, but the optimal amount of fluid to be given has not been established. Observational studies show conflicting results with most indicating possible harm with increasing positive fluid balance, but no studies have been carried out in sepsis patients without shock. From the data provided above in the background section (section 1.2 and 1.3), the current risk/benefit ratio is encouraging restrictive fluid management. As the intervention is very frequent, but not evidence-based and carries potential risks, we consider it to be of great interest for society and patients to perform research in this area. The present trial is a feasibility trial, i.e., a trial assessing the feasibility of the proposed protocol in a clinical setting. Should the trial prove feasible with separation between the two

interventions, a large-scale trial assessing patient important outcomes is intended. Thus, it is the opinion of the steering committee that this study is of great interest and ethically justified.

9.2 Research in sepsis

9.2.1 General considerations

Research in sepsis is ethically challenging for three reasons: 1) patients have impaired cognition due to the sepsis event and can therefore not provide informed consent, 2) patients are in distress when admitted with sepsis, and 3) treatment must be administered quickly (Max. 1 hour as stated by SSC[25]) limiting the possibility of obtaining informed consent from the patient or relatives prior to inclusion.[24] Despite these challenges, there is an ongoing need to conduct research in this specific patient population to improve their outcome, because no other patient groups, who may consent, can be used as a substitute.

Patients with sepsis are temporarily incompetent because of the severe infection with systemic involvement. Taken together, this is an 'acute drug trial' and the patients will be enrolled after proxy consent (from a physician, first trial guardian) according to national law.

International guidelines, such as the revised Declaration of Helsinki[1], European regulations[2], and the Good Clinical Practice guidelines[3], clearly supports research in such populations. For example, the revised Declaration of Helsinki states:

"Research involving subjects who are physically or mentally incapable of giving consent, for example, unconscious patients, may be done only if the physical or mental condition that prevents giving informed consent is a necessary characteristic of the research group. In such circumstances the physician must seek informed consent from the legally authorized representative. If no such representative is available and if the research cannot be delayed, the study may proceed without informed consent provided that the specific reasons for involving subjects with a condition that renders them unable to give informed consent have been stated in the research protocol and the study has been approved by a research ethics committee. Consent to remain in the research must be obtained as soon as possible from the subject or a legally authorised representative."[1]

The current trial will adhere to the revised Declaration of Helsinki as well as all applicable laws and regulatory guidelines.

9.2.2 Danish regulations

Danish law allows research without informed consent from the patient in situation where the following criteria are met[64,65]:

1) The research can only be conducted in the given acute situation
2) The patient is incapable of providing informed consent
3) Consent cannot be obtained from a surrogate given the urgency of the intervention
4) The research specifically involves the patient's current condition
5) There is a possibility of benefit to the patient

The current trial fulfills all the above criteria as described in section 9.2.3 for #1-4 and for #5 in section 9.1 Under these circumstances, research with pharmacological interventions is allowed if the following is obtained[64-66]:

1) Consent is obtained from a designated "legal (trial) guardian" (første "forsøgsværge" in Danish) before enrollment
2) Informed consent is obtained from a surrogate (pårørende in Danish) and/or the patient as soon as feasible

A "legal guardian" is a physician not involved in the research related to the specific patient and who is not in an inferior/superior position to the investigator/sponsor, who should act according to the interest of the research participant.

9.2.3 Regulations in relation to the current trial for patients physically or mentally incapable of giving consent

#1. The research can only be conducted in the given acute situation

Given the high morbidity and mortality of sepsis (see section 1.1.1), clinical trials are highly needed to improve patient outcomes. Sepsis requires ≥2 points in SOFA-score meaning the patient has at least two present organ dysfunctions. There is no other clinical condition that reflects the broad sepsis group and any study aimed to improve outcomes for sepsis patients can therefore only be conducted in this population.

#2. The patient is incapable of providing informed consent

Sepsis is an unpredictable and sudden event; patients have some times been ill for a while, but are, due to rapid disease progression, admitted acutely sick in distress. Sepsis requires ≥2 points in SOFA-score meaning the patient has at least two present organ dysfunctions leaving the patient in a position and mental state,

where they will not be able to provide an informed consent after written and oral information [67,68]. It is therefore impossible to obtain consent prior to or during the acute event for these patients. If the patient is awake, fully conscious and able to understand oral and read written information about the study and therefore capable of providing an informed consent, the patient will not be included in the study.

#3. Consent cannot be obtained from a surrogate given the urgency of the intervention
Sepsis is an acute event that needs administration of antibiotics and fluids within approximately 60 minutes.[25] Given these time frames, it would be impossible to inform the surrogate thoroughly in writing and verbally, allowing for a relative to arrive to support the surrogate as required, provide the required reflection time and obtain consent from a surrogate.

#4. The research specifically involves the patient's current condition
The interventions in this trial are specifically targeted for sepsis patients and if proven effective, will benefit the patient's current condition as well as the sepsis population.

9.3 Procedures

9.3.1 Ethical review committee

The trial will be approved by the regional ethics committee (case number: X).

9.3.2 Trial-specific procedures

The "legal guardian" will be either a physician member of the treating team or a physician on call and available 24/7. The physician might be involved in the clinical care of the patient but will not be involved in trial procedures related to the specific patient. The legal guardian can be involved in trial procedures for other unrelated patients. Through ongoing training and information (see section 3.5.2), the "first trial guardian" will be aware of the trial including the background and significance, inclusion/exclusion criteria, and potential risks and benefits as well as ensuring that the patient can be enrolled in an acute medical trial. This way, the "legal guardian" will be able to make an informed and prompt decision about patient enrollment. The specific details related to the "legal guardian" (i.e., who will be the designated "legal guardian") will be site-specific.

As soon as possible after enrolment (though due to the current COVID-19, see below, this is regarded to be within 10 days of enrollment) consent will be obtained from the participant's next of kin and a second trial guardian. The second trial guardian is also a doctor who is independent of the trial, who has knowledge

of the clinical condition and who is familiar with the trial protocol to such extent that he/she can judge for each patient, if it will be reasonable to enroll the patient in the trial.

When approached, the patient or a surrogate will be informed, verbally and in writing, about the background and significance of the study, inclusion criteria, potential risks and benefits, as well as a brief description of the study protocol. They will be informed that no additional interventions or procedures will be performed after the 24-hour enrollment period. The patient or the surrogate will then provide written informed consent utilizing the informed consent form approved by the ethical review committee. When consent is obtained from participants or a surrogate, information about potential de-identified data sharing will also be included. See Appendix 5 for versions of all consent forms. Consent will be obtained by a physician/other study personnel educated to do so and informed about the study, who has signed for receiving delegated tasks and the procedure requires using and following instructions as described in "*Procedure for afgivelse af mundtlig deltagerinformation, 1. marts 2021, Version 1.0*"; information should be given in calm and undisturbed environment with appropriate time for consideration.

An informed consent includes permission to obtain relevant health information on included patients from the electronic patient journal obtained and accessed by study personnel as well as relevant, controlling authorities (Danish Medicines Agency, Good Clinical Practice and Committee on Health Research Ethics) if necessary.

If SARS-CoV-2 is still a determining factor in the Danish ED at the time of trial enrollment, the following will apply: To minimize the risk of transmission of SARS-CoV-2 between trial staff and the surrogate/next of kin, we will inform and obtain informed consent from the next of kin by telephone. We will contact the next of kin by telephone and arrange a time and date for a telephone conversation with a member of the trial staff (e.g. doctor, research nurse, medical student etc.) who is certified in obtaining informed consent. During this conversation, we will arrange how to send the written information to the next of kin (i.e. e-mail, post). We will encourage the next of kin to read the written information before the next conversation. We will also encourage the next of kin to bring a companion; in this case, the telephone conversation will be held with the telephone on speaker. After we have informed the next of kin about the trial, we will ask the next of kin to return the signed consent form by post.

Participants will be asked for informed consent as soon as possible after they regain the ability to provide consent. For participants, both oral and written information will be given preferably in person. The participant has the right to bring a companion.

If a patient dies before it is possible to obtain consent (we anticipate that approximately 6% will die in-hospital as found in our yet unpublished study), patient data will be included in the trial.[69] If a patient denies

future participation in the trial, no additional data will be collected but all data collected up until the point of withdrawal will be included consistent with Danish law.[70]

The abovementioned trial-specific procedures are in accordance with current trials enrolling patients in Denmark: CLASSIC trial (ClinicalTrials.gov Identifier: NCT03668236) conducted in the ICU and HOT-COVID (ClinicalTrials.gov Identifier: NCT04425031) and COVID-steroid 2-trial (VEK nr. H-20051056, EudraCT nummer 2020-003363-25, ClinicalTrials.gov Identifier: NCT04509973) conducted in all hospital departments with COVID-19 patients.

9.3.3 Insurance

The patients in the study are covered by the Danish patient insurance.[71]

10. MONITORING

10.1 Good Clinical Practice monitoring

The sites will be monitored by the regional Good Clinical Practice monitoring unit affiliated with Aarhus University and Central Denmark Region. A detailed monitoring plan will be developed prior to trial commencement.

11. TIMELINE AND ENROLLMENT

11.1 Timeline

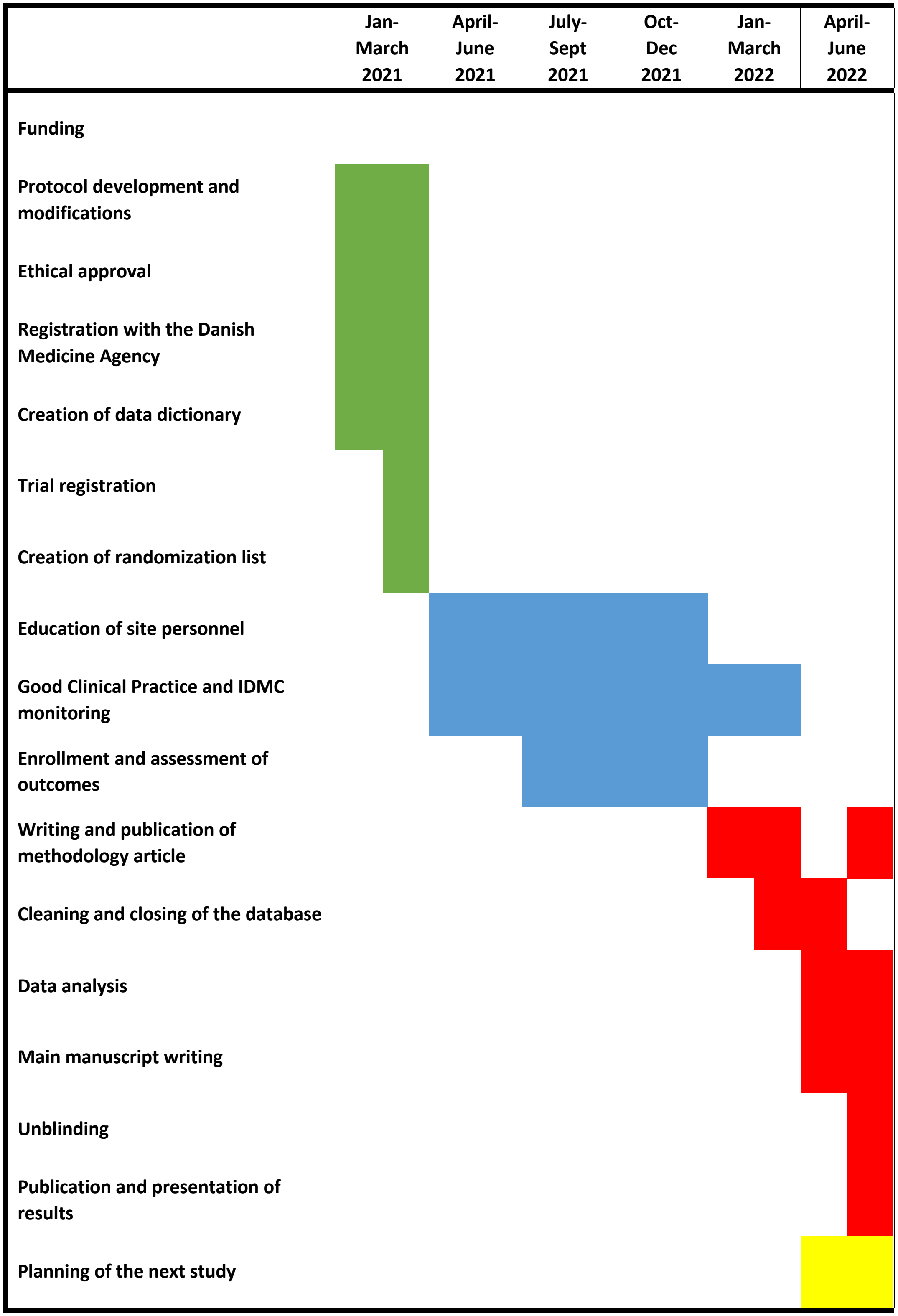

11.2 Feasibility

Data from 2020 from two of the participating hospitals are provided in Table 1. As illustrated, we expect approximately 64 patients to be eligible for enrollment each week from the two included sites. Given the acuity of sepsis and the busy environment in the emergency departments, we expect inclusion to last 2-3 months, with an enrollment rate as low as 35% and only day-time-enrollment if necessary. The number of sepsis patients at Regional Hospital Viborg is unknown, but excepted to be equal to or larger than Regional Hospital Randers due to its larger total number of hospital admissions.

Table 1. 2020 data on sepsis patients from three participating sites during six weeks, Jan 20-March 2 2020

Hospital	Number of sepsis patients	Sepsis patients admitted > 24 hours	Average number of sepsis patients per week admitted > 24 hours
Aarhus University Hospital	337	275	46
Regional Hospital Randers	203	107	18
**Total for the two included sites**	**540**	**382**	**64**

11.3 Enrollment

Enrollment at each site will be continuously monitored by the sponsor, principal site investigators, and the research nurse. Formal reports outlining the number of sepsis patients and the proportion of those enrolled at each site will be shared with the steering committee monthly.

12. PUBLICATION PLAN

Two manuscripts are planned from the current trial. Results will be published regardless of outcome; positive, negative and inconclusive results will be published. Prior to enrollment of the last patient, a methodology article will be published including a detailed description of the trial and the statistical analysis plan. The second and primary manuscript will include the main results including pre-defined primary and secondary outcomes. The manuscript will adhere to the CONSORT guidelines.[61,62] The principal investigator (sponsor investigator) will be the first and corresponding author. Additional authorship will follow

authorship guidelines from the International Committee of Medical Journal Editors[72] and will include members of the steering committee and one investigator per sites that have recruited at least one participant. In addition, as a guideline, sites enrolling > 20 patients will be entitled one additional author and sites enrolling > 40 patients two additional authors in addition to the site investigator and members of the steering committee. This will only apply to the main manuscript of the study. The main results will be presented at an international conference. The trial results will be shared with participating sites and via press releases but not directly with the participating patients but linked to via the study web-site. Study findings will be published irrespective of the results. Trial results will be uploaded in EudraCT as soon as possible and at the latest within a year after trial completion. Data will be publicly available at clinicaltrialsregister.eu after this upload.

13. DATA SHARING

Six months after the publication of the last results, all de-identified individual patient data will be made available for data sharing.[73] Procedures, including re-coding of key variables, will be put in place to allow for complete de-identification of the data. Data will be completely anonymized according to Danish law.

All relevant trial-related documents, including the protocol, data dictionary, and the main statistical code, will be shared along with the data. There will be no predetermined end date for the data sharing. Data will be available for any research purpose to all interested parties who have approval from an independent review committee and who have a methodological sound proposal as determined by the steering committee of the current trial. Only the methodological qualities and not the purpose or objective of the proposal will be considered. Interested parties will be able to request the data by contacting the sponsor investigator. Authorship of potentially additional publications emerging from the shared data will follow standard authorship guidelines from the International Committee of Medical Journal Editors[72] and might or might not include authors from the steering committee depending on the nature of their involvement.

14. FUNDING

Funding for the trial is provided by Carl and Ellen Hertz foundation (DKK 15,000), Frimodt-Heineke Foundation (DKK 25,000), Ruth & Holger Hesses Memorial Fund (DKK 60,000), Health Research Foundation of Central Denmark Region (DKK 215,000), “Akutpuljen” Central Denmark Region (DKK 582,000), and Aarhus University (salary for primary investigator, DKK 1.5 mio). Funding is administered at the Research Center for Emergency Medicine, Central Denmark Region and is used for salary support, monitoring, and additional

operational expenses. Additional funding will be applied for at various private and public foundations. The funding agencies or any pharmaceutical companies will have no role in the design and conduct of the study; collection, management, analysis, and interpretation of the data; preparation, review, or approval of the manuscript; or the decision to submit the manuscript for publication. None of the members of the steering committee have any financial disclosures or financial connections to any of the foundations.

15. TASKS AND RESPONSIBILITIES

Sponsor investigator, sponsor, and coordinating investigator: Overall responsibility for protocol development, funding, budget overview, data dictionary development, ethical approval, trial registration, daily management, trial oversight, contact to the pharmacy, contact to Good Clinical Practice monitoring unit and the data and safety monitoring board, assessment of overall recruitments and education, potential recruitment of additional sites, data analysis, and dissemination and presentation of results

Steering committee: Protocol development, funding, budget overview, data dictionary development, trial oversight, dissemination of results, responsibilities as sponsor investigator for short time periods

Primary Site investigators: Responsible for site-specific screening and enrollment, evaluation of eligible patients not included, education of personnel at participating sites, reporting of site-specific issues or challenges to the principal investigator, participant consent for data collection

Research nurse/medical students: Daily management and screening logs, education of personnel at participating sites, contact to Good Clinical Practice monitoring unit, data dictionary development, data entry and management, patient follow-up,

Clinical team: enrollment of patients, obtaining informed consent after being trained in doing this, deliver care in adherence to the trial protocol, register data on paper-CRF

Good Clinical Practice-unit: See section 10.1. and Appendix 6 for monitoring plan

References

1. World Medical Association. World Medical Association Declaration of Helsinki: ethical principles for medical research involving human subjects. *JAMA.* 2013;310(20):2191-2194.
2. The European Parliament and the Council of the European Unit. Clinical Trials Regulation (CTR) EU No 536/2014. 2014; https://ec.europa.eu/health/sites/health/files/files/eudralex/vol-1/reg_2014_536/reg_2014_536_en.pdf. Accessed July 18, 2017.
3. ICH Harmonised Tripartite Guideline. Integrated Addendum To ICH E6(R1): Guideline For Good Clinical Practice E6(R2). 2016; http://www.ich.org/fileadmin/Public_Web_Site/ICH_Products/Guidelines/Efficacy/E6/E6_R2__Step_4.pdf. Accessed June 30, 2017.
4. ICH Harmonised Tripartite Guideline. General Considerations for Clinical Trials E8. 1997; http://www.ich.org/fileadmin/Public_Web_Site/ICH_Products/Guidelines/Efficacy/E8/Step4/E8_Guideline.pdf. Accessed June 30, 2017.
5. ICH Harnomised Tripartite Guidelines. Statistical Principles for Clinical Trials. 1998; http://www.ich.org/fileadmin/Public_Web_Site/ICH_Products/Guidelines/Efficacy/E9/Step4/E9_Guideline.pdf. Accessed June 30, 2017.
6. Chan AW, Tetzlaff JM, Altman DG, et al. SPIRIT 2013 statement: defining standard protocol items for clinical trials. *Ann Intern Med.* 2013;158(3):200-207.
7. Chan AW, Tetzlaff JM, Gotzsche PC, et al. SPIRIT 2013 explanation and elaboration: guidance for protocols of clinical trials. *BMJ.* 2013;346:e7586.
8. Henriksen DP, Laursen CB, Jensen TG, Hallas J, Pedersen C, Lassen AT. Incidence rate of community-acquired sepsis among hospitalized acute medical patients-a population-based survey. *Crit Care Med.* 2015;43(1):13-21.
9. Wang HE, Shapiro NI, Angus DC, Yealy DM. National estimates of severe sepsis in United States emergency departments. *Crit Care Med.* 2007;35(8):1928-1936.
10. Capp R, Horton CL, Takhar SS, et al. Predictors of patients who present to the emergency department with sepsis and progress to septic shock between 4 and 48 hours of emergency department arrival. *Crit Care Med.* 2015;43(5):983-988.
11. Glickman SW, Cairns CB, Otero RM, et al. Disease progression in hemodynamically stable patients presenting to the emergency department with sepsis. *Acad Emerg Med.* 2010;17(4):383-390.
12. Arnold RC, Sherwin R, Shapiro NI, et al. Multicenter observational study of the development of progressive organ dysfunction and therapeutic interventions in normotensive sepsis patients in the emergency department. *Acad Emerg Med.* 2013;20(5):433-440.
13. Jessen MK, Mackenhauer J, Hvass AM, Heide-Jorgensen U, Christiansen CF, Kirkegaard H. Predictors of intensive care unit transfer or death in emergency department patients with suspected infection. *Eur J Emerg Med.* 2015;22(3):176-180.
14. Paoli CJ, Reynolds MA, Sinha M, Gitlin M, Crouser E. Epidemiology and Costs of Sepsis in the United States-An Analysis Based on Timing of Diagnosis and Severity Level. *Crit Care Med.* 2018.
15. Whiles BB, Deis AS, Simpson SQ. Increased Time to Initial Antimicrobial Administration Is Associated With Progression to Septic Shock in Severe Sepsis Patients. *Crit Care Med.* 2017;45(4):623-629.
16. Marik PE, Linde-Zwirble WT, Bittner EA, Sahatjian J, Hansell D. Fluid administration in severe sepsis and septic shock, patterns and outcomes: an analysis of a large national database. *Intensive Care Med.* 2017;43(5):625-632.
17. Perner A, Lassen AT, Schierbeck J, Storgaard M, Reiter N, Benfield T. [Disease burden and definition of sepsis in adults]. *Ugeskrift for laeger.* 2018;180(15).
18. Hammond NE, Finfer SR, Li Q, et al. Health-related quality of life in survivors of septic shock: 6-month follow-up from the ADRENAL trial. *Intensive Care Med.* 2020;46(9):1696-1706.

19. Winters BD, Eberlein M, Leung J, Needham DM, Pronovost PJ, Sevransky JE. Long-term mortality and quality of life in sepsis: a systematic review. *Crit Care Med.* 2010;38(5):1276-1283.
20. Prescott HC, Langa KM, Liu V, Escobar GJ, Iwashyna TJ. Increased 1-year healthcare use in survivors of severe sepsis. *American journal of respiratory and critical care medicine.* 2014;190(1):62-69.
21. Singer M, Deutschman CS, Seymour CW, et al. The Third International Consensus Definitions for Sepsis and Septic Shock (Sepsis-3). *Jama.* 2016;315(8):801-810.
22. Incidence of severe sepsis and septic shock in German intensive care units: the prospective, multicentre INSEP study. *Intensive Care Med.* 2016;42(12):1980-1989.
23. Jessen MK, Mackenhauer J, Hvass AM, et al. Prediction of bacteremia in the emergency department: an external validation of a clinical decision rule. *Eur J Emerg Med.* 2016;23(1):44-49.
24. Rhodes A, Evans LE, Alhazzani W, et al. Surviving Sepsis Campaign: International Guidelines for Management of Sepsis and Septic Shock: 2016. *Crit Care Med.* 2017;45(3):486-552.
25. Levy MM, Evans LE, Rhodes A, et al. The Surviving Sepsis Campaign Bundle: 2018 Update
CE: Managing Sepsis and Septic Shock: Current Guidelines and Definitions. *Crit Care Med.* 2018;46(6):997-1000.
26. Harris T, Coats TJ, Elwan MH. Fluid therapy in the emergency department: an expert practice review. *Emergency medicine journal : EMJ.* 2018;35(8):511-515.
27. Myburgh JA, Mythen MG. Resuscitation fluids. *N Engl J Med.* 2013;369(13):1243-1251.
28. Prowle JR, Kirwan CJ, Bellomo R. Fluid management for the prevention and attenuation of acute kidney injury. *Nature reviews Nephrology.* 2014;10(1):37-47.
29. Legrand M, Dupuis C, Simon C, et al. Association between systemic hemodynamics and septic acute kidney injury in critically ill patients: a retrospective observational study. *Critical care (London, England).* 2013;17(6):R278.
30. van Genderen ME, Klijn E, Lima A, et al. Microvascular perfusion as a target for fluid resuscitation in experimental circulatory shock. *Crit Care Med.* 2014;42(2):e96-e105.
31. Dellinger RP, Carlet JM, Masur H, et al. Surviving Sepsis Campaign guidelines for management of severe sepsis and septic shock. *Crit Care Med.* 2004;32(3):858-873.
32. Hjortrup PB, Haase N, Wetterslev J, Perner A. Associations of Hospital and Patient Characteristics with Fluid Resuscitation Volumes in Patients with Severe Sepsis: Post Hoc Analyses of Data from a Multicentre Randomised Clinical Trial. *PLoS One.* 2016;11(5):e0155767.
33. Angus DC, Barnato AE, Bell D, et al. A systematic review and meta-analysis of early goal-directed therapy for septic shock: the ARISE, ProCESS and ProMISe Investigators. *Intensive Care Med.* 2015;41(9):1549-1560.
34. Ueyama H, Kiyonaka S. Predicting the Need for Fluid Therapy-Does Fluid Responsiveness Work? *Journal of intensive care.* 2017;5:34.
35. Malbrain ML, Marik PE, Witters I, et al. Fluid overload, de-resuscitation, and outcomes in critically ill or injured patients: a systematic review with suggestions for clinical practice. *Anaesthesiol Intensive Ther.* 2014;46(5):361-380.
36. Seymour CW, Gesten F, Prescott HC, et al. Time to Treatment and Mortality during Mandated Emergency Care for Sepsis. *N Engl J Med.* 2017;376(23):2235-2244.
37. Boyd JH, Forbes J, Nakada TA, Walley KR, Russell JA. Fluid resuscitation in septic shock: a positive fluid balance and elevated central venous pressure are associated with increased mortality. *Crit Care Med.* 2011;39(2):259-265.
38. Kelm DJ, Perrin JT, Cartin-Ceba R, Gajic O, Schenck L, Kennedy CC. Fluid overload in patients with severe sepsis and septic shock treated with early goal-directed therapy is associated with increased acute need for fluid-related medical interventions and hospital death. *Shock (Augusta, Ga).* 2015;43(1):68-73.
39. Wu X, Hu Z, Yuan H, Chen L, Li Y, Zhao C. Fluid Resuscitation and Markers of Glycocalyx Degradation in Severe Sepsis. *Open Med (Wars).* 2017;12:409-416.

40. Malbrain M, Van Regenmortel N, Saugel B, et al. Principles of fluid management and stewardship in septic shock: it is time to consider the four D's and the four phases of fluid therapy. *Ann Intensive Care.* 2018;8.
41. Sethi M, Owyang CG, Meyers C, Parekh R, Shah KH, Manini AF. Choice of resuscitative fluids and mortality in emergency department patients with sepsis. *Am J Emerg Med.* 2018;36(4):625-629.
42. Shaw AD, Raghunathan K, Peyerl FW, Munson SH, Paluszkiewicz SM, Schermer CR. Association between intravenous chloride load during resuscitation and in-hospital mortality among patients with SIRS. *Intensive Care Med.* 2014;40(12):1897-1905.
43. Byrne L, Obonyo NG, Diab SD, et al. Unintended Consequences: Fluid Resuscitation Worsens Shock in an Ovine Model of Endotoxemia. *American journal of respiratory and critical care medicine.* 2018;198(8):1043-1054.
44. Marik PE, Monnet X, Teboul JL. Hemodynamic parameters to guide fluid therapy. *Ann Intensive Care.* 2011;1(1):1.
45. Hjortrup PB, Haase N, Bundgaard H, et al. Restricting volumes of resuscitation fluid in adults with septic shock after initial management: the CLASSIC randomised, parallel-group, multicentre feasibility trial. *Intensive Care Med.* 2016;42(11):1695-1705.
46. Macdonald SPJ, Keijzers G, Taylor DM, et al. Restricted fluid resuscitation in suspected sepsis associated hypotension (REFRESH): a pilot randomised controlled trial. *Intensive Care Med.* 2018;44(12):2070-2078.
47. Meyhoff TS, Møller MH, Hjortrup PB, Cronhjort M, Perner A, Wetterslev J. Lower vs Higher Fluid Volumes During Initial Management of Sepsis: A Systematic Review With Meta-Analysis and Trial Sequential Analysis. *Chest.* 2020;157(6):1478-1496.
48. Hjortrup PB, Haase N, Wetterslev J, et al. Effects of fluid restriction on measures of circulatory efficacy in adults with septic shock. *Acta Anaesthesiol Scand.* 2017;61(4):390-398.
49. Self WH, Semler MW, Bellomo R, et al. Liberal Versus Restrictive Intravenous Fluid Therapy for Early Septic Shock: Rationale for a Randomized Trial. *Ann Emerg Med.* 2018.
50. Macdonald SPJ, Taylor DM, Keijzers G, et al. REstricted Fluid REsuscitation in Sepsis-associated Hypotension (REFRESH): study protocol for a pilot randomised controlled trial. *Trials.* 2017;18(1):399.
51. Perner A, Gordon AC, Angus DC, et al. The intensive care medicine research agenda on septic shock. *Intensive Care Med.* 2017;43(9):1294-1305.
52. Kernan WN, Viscoli CM, Makuch RW, Brass LM, Horwitz RI. Stratified randomization for clinical trials. *J Clin Epidemiol.* 1999;52(1):19-26.
53. Ait-Oufella H, Lemoinne S, Boelle PY, et al. Mottling score predicts survival in septic shock. *Intensive Care Med.* 2011;37(5):801-807.
54. Levy MM, Evans LE, Rhodes A. The Surviving Sepsis Campaign Bundle: 2018 update. *Intensive Care Med.* 2018;44(6):925-928.
55. Wacharasint P, Nakada TA, Boyd JH, Russell JA, Walley KR. Normal-range blood lactate concentration in septic shock is prognostic and predictive. *Shock (Augusta, Ga).* 2012;38(1):4-10.
56. Webb AL, Kramer N, Rosario J, et al. Delta Lactate (Three-hour Lactate Minus Initial Lactate) Prediction of In-hospital Death in Sepsis Patients. *Cureus.* 2020;12(4):e7863.
57. Jouffroy R, Saade A, Tourtier JP, et al. Skin mottling score and capillary refill time to assess mortality of septic shock since pre-hospital setting. *Am J Emerg Med.* 2019;37(4):664-671.
58. Nichol G, Powell JL, Emerson S. On coenrollment in clinical resuscitation studies: review and experience from randomized trials. *Resuscitation.* 2010;81(7):792-795.
59. Kellum JA, Lameire N. Diagnosis, evaluation, and management of acute kidney injury: a KDIGO summary (Part 1). *Critical care (London, England).* 2013;17(1):204.

60. Schmidt M, Schmidt SA, Sandegaard JL, Ehrenstein V, Pedersen L, Sorensen HT. The Danish National Patient Registry: a review of content, data quality, and research potential. *Clin Epidemiol.* 2015;7:449-490.
61. Schulz KF, Altman DG, Moher D, Group C. CONSORT 2010 statement: updated guidelines for reporting parallel group randomised trials. *BMJ.* 2010;340:c332.
62. Moher D, Hopewell S, Schulz KF, et al. CONSORT 2010 explanation and elaboration: updated guidelines for reporting parallel group randomised trials. *BMJ.* 2010;340:c869.
63. Harris PA, Taylor R, Thielke R, Payne J, Gonzalez N, Conde JG. Research electronic data capture (REDCap)--a metadata-driven methodology and workflow process for providing translational research informatics support. *Journal of biomedical informatics.* 2009;42(2):377-381.
64. Lov om videnskabsetisk behandling af sundhedsvidenskabelige forskningsprojekter [Danish]. 2011; https://www.retsinformation.dk/forms/r0710.aspx?id=137674 - Not1. Accessed July 7, 2017.
65. Vejledning om akutte forsøg [Danish]. 2017; http://www.nvk.dk/emner/akutte-forsoeg/vejledning-om-akutte-forsoeg - 1. Accessed July 7, 2017.
66. Lov om kliniske forsøg med lægemidler [Danish]. 2016; https://www.retsinformation.dk/Forms/r0710.aspx?id=180117. Accessed July 7, 2017.
67. Le Dorze M, Huche F, Coelembier C, Rabuel C, Payen D. Impact of fluid challenge increase in cardiac output on the relationship between systemic and cerebral hemodynamics in severe sepsis compared to brain injury and controls. *Ann Intensive Care.* 2018;8(1):74.
68. Tauber SC, Djukic M, Gossner J, Eiffert H, Brück W, Nau R. Sepsis-associated encephalopathy and septic encephalitis: an update. *Expert Rev Anti Infect Ther.* 2020:1-17.
69. Ministeriet for Sundhed og Forebyggelse. Letter to Den Nationale Videnskabsetiske Komité dated February 28th, 2014 regarding "Anvendelse af data i akutforsøg" [Danish].
70. Lov om ændring af lov om videnskabsetisk behandling af sundhedsvidenskabelige forskningsprojekter [Danish]. 2012; https://www.retsinformation.dk/forms/r0710.aspx?id=142351. Accessed July 13, 2017.
71. Bekendtgørelse af lov om klage- og erstatningsadgang inden for sundhedsvæsenet [Danish]. 2011; https://www.retsinformation.dk/Forms/r0710.aspx?id=138893. Accessed July 13, 2017.
72. International Committee of Medical Journal Editors. Defining the Role of Authors and Contributors. 2017; http://www.icmje.org/recommendations/browse/roles-and-responsibilities/defining-the-role-of-authors-and-contributors.html. Accessed July 6, 2017.
73. Taichman DB, Sahni P, Pinborg A, et al. Data Sharing Statements for Clinical Trials - A Requirement of the International Committee of Medical Journal Editors. *N Engl J Med.* 2017;376(23):2277-2279.
74. Seymour CW, Liu VX, Iwashyna TJ, et al. Assessment of Clinical Criteria for Sepsis: For the Third International Consensus Definitions for Sepsis and Septic Shock (Sepsis-3). *Jama.* 2016;315(8):762-774.
75. Shao IY, Elkind MSV, Boehme AK. Risk Factors for Stroke in Patients With Sepsis and Bloodstream Infections. *Stroke.* 2019;50(5):1046-1051.
76. Court O, Kumar A, Parrillo JE, Kumar A. Clinical review: Myocardial depression in sepsis and septic shock. *Critical care (London, England).* 2002;6(6):500-508.
77. Nandhabalan P, Ioannou N, Meadows C, Wyncoll D. Refractory septic shock: our pragmatic approach. *Critical care (London, England).* 2018;22(1):215.
78. Uchino S, Kellum JA, Bellomo R, et al. Acute renal failure in critically ill patients: a multinational, multicenter study. *Jama.* 2005;294(7):813-818.
79. Matuschak GM, Lechner AJ. Acute lung injury and the acute respiratory distress syndrome: pathophysiology and treatment. *Mo Med.* 2010;107(4):252-258.

Appendices

Appendix 1: Conflict of interest disclosures for the steering committee members

Marie K Jessen

Industry:

- None

Other:

- None

Lars W. Andersen

Industry:

- None

Other:

- None

Hans Kirkegaard

Industry:

- None

Other:

- None

Jens Aage Kølsen Petersen

Industry:

- None

Other:

- None

Anders Perner

Industry:

- Grant from Pfizer, Denmark
- Grant from Novo Nordisk Foundation, Denmark

Other:

- Sponsor and primary investigator of the CLASSIC trial (ClinicalTrials.gov Identifier: NCT03668236)

Ranva Espegård Hassel

Industry:

- None

Other:

- None

Wazhma Hayeri

Industry:

- None

Other:

- None

Peter Kristensen

Industry:

- None

Other:

- None

Appendix 2: Sequential organ failure assessment (SOFA) score

The Sequential organ failure assessment (SOFA)-score is in the current study defined in accordance with The Third International Consensus Definitions for Sepsis and Septic Shock (Sepsis-3)[21,74]

Sequential [Sepsis-related] Organ-Failure Assessment Score adapted from Sepsis-3[21]					
	**Score**				
**System**	**0**	**1**	**2**	**3**	**4**
**Respiration**					
PaO_2/FiO_2, kPa	≥ 53.3	< 53.3	< 40	< 26.7 with respiratory support	< 13.3 with respiratory support
**Coagulation**					
Platelets, x 10^3/µL	≥ 150	< 150	< 100	< 50	< 20
**Liver**					
Billirubin, µmol/L	< 20	20-32	33-101	102-204	> 204
**Cardiovascular**	MAP ≥ 70 mmHg	MAP < 70 mmHg	Dopamine<5 or dobutamine (any dose) [a]	Dopamine 5.1-15 or epinephrine ≤0.1 or norepinephrine ≤0.1 [a]	Dopamine > 15 or epinephrine >0.1 or norepinephrine >0.1 [a]
**Central nervous system**					
Glasgow Coma Scale Score	15	13-14	10-12	6-9	<6
**Renal**					
Creatinine, µmol/L	< 110	110-170	171-299	300-400	> 440
Urine output, mL/d				< 500	< 200

The table shows the scoring of organ dysfunctions with the Sequential [Sepsis-related] Organ-Failure Assesment Score. The final SOFA-score is calculated summing all sub-scores from each organ system. Only definitions with units used in Danish emergency departments are shown.
Abbreviations: PaO_2: Partial pressure of oxygen, FiO_2: Fraction of inspired oxygen, MAP: Mean arterial pressure
[a] Catecholamine doses are given as µg/kg/min for at least 1 hour
[b] Glasgow Coma Scale Scores range from 3-15: Higher scores indicate better neurological function

Appendix 3: Procedure for reporting serious adverse events and serious adverse reactions from randomization to day 7 in REFACED Sepsis

Flow chart for reporting serious adverse events (SAEs) or reactions (SARs) from randomization until day 7 for conditions listed below

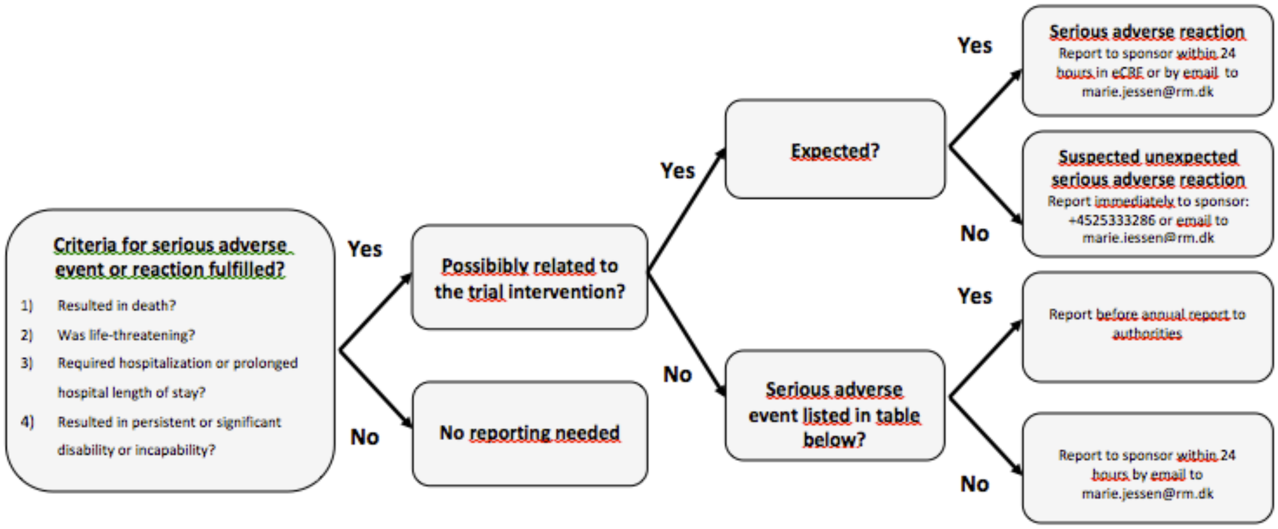

The listed serious adverse events do not have to be reported to the sponsor within 24 hours of occurrence if adjudicated not to be related to the intervention and expected in the patient population of sepsis patients.

List of SAEs that do NOT have to be reported to the sponsor within 24 hours of occurrence

- New ischemic events defined as:
  - Cerebral ischemia as any form of cerebral ischemia on a CT- or MRI scan[75]
  - Acute myocardial ischemia defined as participant with acute myocardial infarction (ST-elevation myocardial infarction or non-ST elevation myocardial infarction)[76] or unstable angina pectoris according to the criteria in the clinical setting (e.g. elevated biomarkers, ischemic signs on ECG, and clinical presentation)
  - Intestinal ischemia defined as ischemia verified with endoscopy, surgery, or CT-angiography.
  - Limb ischemia defined as clinical signs and need of vascular intervention, amputation, or initiation/increased antithrombotic treatment.
- Heart failure or cardiogenic shock [77] or cardiac arrest/death[14-16]
- New onset of severe acute kidney injury (modified KDIGO3)[78]
- Hypervolemia/overhydration, pulmonary edema[79]

All of the above SAEs should be reported to sponsor before final report to the authorities.

Appendix 4: Product summaries of used fluids

See elsewhere in the application

Appendix 5: Informed consent forms

(S9)

Stedfortrædende samtykke fra forsøgsværgen til akut lægemiddelforsøg.

Forskningsprojektets titel: Restriktiv vs. vanlig væskebehandling af patienter med sepsis

(Engelsk: Restrictive Fluid Administration vs. Standard of Care in Emergency Department Sepsis Patients - a Multicenter, Randomized Clinical Feasibility Trial (REFACED Sepsis))

Erklæring fra forsøgsværgen (en uafhængig læge):

Jeg erklærer hermed, at jeg har fået skriftlig information om det konkrete forskningsprojekt samt oplysning om forsøgspersonens tilstand. Jeg er uafhængig af den forsøgsansvarliges interesser og af interesser i forskningsprojekter i øvrigt og giver – som varetager af forsøgspersonens interesser – samtykke til, at

____ (forsøgspersonens navn)

deltager i forskningsprojektet.

Navnet på forsøgsværgen: ____

Dato: ________________ Klokkeslæt: ________________

Underskrift: ____

Erklæring fra den, der afgiver information:

Jeg erklærer, at forsøgsværgen har fået skriftlig information om det konkrete forskningsprojekt samt oplysning om forsøgspersonens tilstand.

Navnet på den, der har afgivet information:

Dato: ________________ Underskrift: ____

Projektidentifikation:

EudraCT number: 2021-000224-35

REFACED Sepsis - version 1.0 – 1. marts 2021
Samtykke givet jf. informationsmateriale v.1.0

(S7)

Stedfortrædende samtykke til deltagelse i et sundhedsvidenskabeligt forskningsprojekt.

Forskningsprojektets titel: Restriktiv vs. vanlig væskebehandling af patienter med sepsis

Erklæring fra den person, som afgiver stedfortrædende samtykke:

Jeg har fået skriftlig og mundtlig information og jeg ved nok om formål, metode, fordele og ulemper til at give mit samtykke.

Jeg ved, at det er frivilligt at deltage, og at jeg altid kan trække mit samtykke tilbage uden at forsøgspersonen mister sine nuværende eller fremtidige rettigheder til behandling.

Jeg giver samtykke til, at ____ (forsøgspersonens navn) deltager i forskningsprojektet og jeg har fået en kopi af dette samtykkeark samt en kopi af den skriftlige information om projektet til eget brug.

Oplysning om min tilknytning, som pårørende, til forsøgspersonen:

____

Navnet på den person, der giver stedfortrædende samtykke: ______________________________

Dato: ________________ Klokkeslæt: ________________

Underskrift: ____

Ønskes information om forskningsprojektets resultat samt eventuelle konsekvenser for forsøgspersonen?:

Ja _____ (sæt x) Nej _____ (sæt x)

Erklæring fra den, der afgiver informationen til pårørende:

Jeg erklærer, at der er afgivet mundtlig og skriftlig information om forsøget.

Navnet på den, der har afgivet information:

Dato: ________________ Underskrift: ____

____

Stedfortrædende samtykke fra forsøgsværgen (en uafhængig læge):

Dato: ________________ Klokkeslæt: ________________

Underskrift: ____

Erklæring fra den, der afgiver informationen til forsøgsværgen (en uafhængig læge):

Jeg erklærer, at der er afgivet mundtlig og skriftlig information om forsøget.

Navnet på den, der har afgivet information:

Dato: ________________ Underskrift: ____

Projektidentifikation:
EudraCT number: 2021-000224-35

REFACED Sepsis – version 1.0 -1. marts 2021
Samtykke givet jf. informationsmateriale v.1.0

(S1)

Informeret samtykke til deltagelse i et sundhedsvidenskabeligt forskningsprojekt.

Forskningsprojektets titel: Restriktiv vs. vanlig væskebehandling af patienter med sepsis

Erklæring fra forsøgspersonen:

Jeg har fået skriftlig og mundtlig information og jeg ved nok om formål, metode, fordele og ulemper til at sige ja til at deltage.

Jeg ved, at det er frivilligt at deltage, og at jeg altid kan trække mit samtykke tilbage uden at miste mine nuværende eller fremtidige rettigheder til behandling.

Jeg giver samtykke til, at deltage i forskningsprojektet, og har fået en kopi af dette samtykkeark samt en kopi af den skriftlige information om projektet til eget brug.

Forsøgspersonens navn: ____

Dato: ________________ Klokkeslæt: ________________

Underskrift: ____

Ønsker du at blive informeret om forskningsprojektets resultat samt eventuelle konsekvenser for dig?:

Ja _____ (sæt x) Nej _____ (sæt x)

Erklæring fra den, der afgiver information:

Jeg erklærer, at forsøgspersonen har modtaget mundtlig og skriftlig information om forsøget.

Efter min overbevisning er der givet tilstrækkelig information til, at der kan træffes beslutning om deltagelse i forsøget.

Navnet på den, der har afgivet information:

Dato: ________________ Underskrift: ____

Projektidentifikation:
EudraCT number: 2021-000224-35

REFACED Sepsis - version 1.0 – 1. marts 2021
Samtykke givet jf. informationsmateriale v.1.0

Appendix 6: Good-clinical-practice monitoring plan

Will be developed prior to trial commencement

Appendix 7: Draft of CONSORT flow diagram

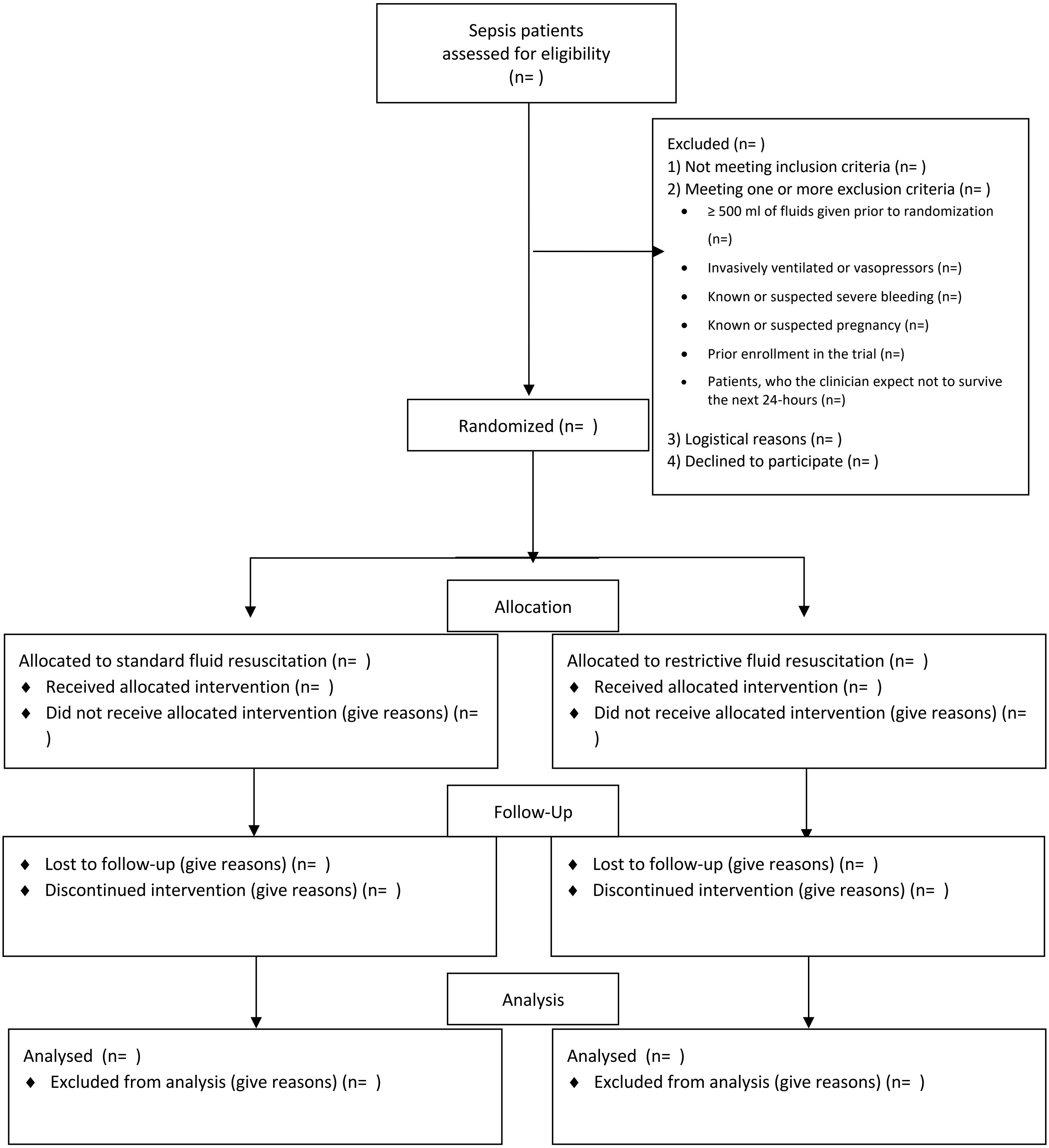

Supplement: Supplementary file 1 — Additional file 1. [file 40814_2022_1034_MOESM1_ESM.pdf]
